# Detecting antibiotic resistance: classical, molecular, advanced bioengineering, and AI-enhanced approaches

**DOI:** 10.3389/fmicb.2025.1673343

**Published:** 2025-10-03

**Authors:** Alexandru Constantin Aldea, Filofteia Camelia Diguṭă, Oriana Presacan, Cătălina Voaideṣ, Radu Cristian Toma, Florentina Matei

**Affiliations:** ^1^Faculty of Biotechnologies, University of Agronomic Sciences and Veterinary Medicine of Bucharest, Bucharest, Romania; ^2^Faculty of Electronics, Telecommunications, and Information Technology, National University of Science and Technology Politehnica Bucharest, Bucharest, Romania; ^3^Faculty of Food Industry and Tourism, Transilvania University of Braşov, Braşov, Romania

**Keywords:** antibiotic resistance, pathogens, detection methods, multidrug resistance, ESKAPE, nanotechnological platforms, artificial intelligence, machine learning

## Abstract

Antibiotic resistance continues to erode the effectiveness of modern medicine, creating an urgent demand for rapid and reliable diagnostic solutions. Conventional diagnostic approaches, including culture-based susceptibility testing, remain the clinical reference standard but are constrained by lengthy turnaround times and limited sensitivity for early detection. In recent years, significant progress has been made with molecular and spectrometry-based methods, such as PCR and next-generation sequencing, MALDI-TOF MS, Raman and FTIR spectroscopy, alongside emerging CRISPR-based platforms. Complementary innovations in biosensors, microfluidics, and artificial intelligence further expand the diagnostic landscape, enabling faster, more sensitive, and increasingly portable assays. This review examines both established and emerging technologies for detecting antibiotic resistance, outlining their respective strengths, limitations, and potential roles across diverse settings. By synthesizing current advances and highlighting future opportunities, this review emphasizes complementarities among detection strategies and their potential integration into practical diagnostic frameworks, including in resource-limited settings.

## 1 Introduction

Infectious diseases have shaped human history, causing devastating pandemics and influencing medical advancements. The introduction of antibiotics in the early 1900s dramatically reduced mortality from bacterial infections and revolutionized medicine (Hutchings et al., 2019). However, this triumph has been overshadowed by the rapid evolution of antibiotic resistance, which now threatens decades of progress and is responsible for more than 1.14 million deaths annually, with projections exceeding 8 million by 2050 if urgent measures are not implemented (Li Z. et al., 2024; Naghavi et al., 2024; Compaoré et al., 2024; Frieri et al., 2017; Maragakis et al., 2008).

Particularly concerning are the so-called ESKAPE pathogens: *Enterococcus faecium, Staphylococcus aureus, Klebsiella pneumoniae, Acinetobacter baumannii, Pseudomonas aeruginosa*, and *Enterobacter* species, which account for a large share of healthcare-associated infections (Daruka et al., 2025; Miller and Arias, 2024). The WHO's 2024 Bacterial Priority Pathogen List classifies carbapenem-resistant *A. baumannii* and third-generation cephalosporin- or carbapenem-resistant Enterobacterales as critical priority pathogens, while vancomycin-resistant *E. faecium*, carbapenem-resistant *P. aeruginosa*, and methicillin-resistant *S. aureus* (MRSA) are listed as high priority, reflecting their major clinical impact and urgent need for new treatments (World Health Organization, 2024). Local epidemiological studies corroborate this threat: for example, in a 2025 surgical-site infection study in Ethiopia, 84.4% of ESKAPE isolates were multidrug-resistant (MDR), with *A. baumannii* showing 100% MDR rates (Seid et al., 2025). Moreover, a “One Health” systematic review in Africa (Khasapane et al., 2024) highlights the widespread occurrence of these pathogens in humans, animals, food, and environmental reservoirs, underscoring their persistence and dissemination potential.

The emergence of antibiotic resistance is a multifaceted issue driven by various factors. One of the most widely recognized and publicized causes is the overuse of antibiotics, which exerts a strong selective pressure by killing susceptible bacteria and allowing resistant strains to thrive. These resistant bacteria not only proliferate but also disseminate their resistance determinants through horizontal gene transfer (HGT), thereby accelerating the spread of resistance genes (Kunhikannan et al., 2021; Tripathi and Tripathi, 2017). However, resistance is not solely a consequence of modern antibiotic use. Notably, β-lactam, tetracycline, and glycopeptide resistance genes have been identified in 30,000-year-old permafrost sediments, suggesting that resistance is an ancient phenomenon that predates clinical antibiotic application (D'Costa et al., 2011). Nevertheless, human activities, particularly in healthcare and agriculture, have dramatically accelerated its global dissemination.

**Figure 1:**
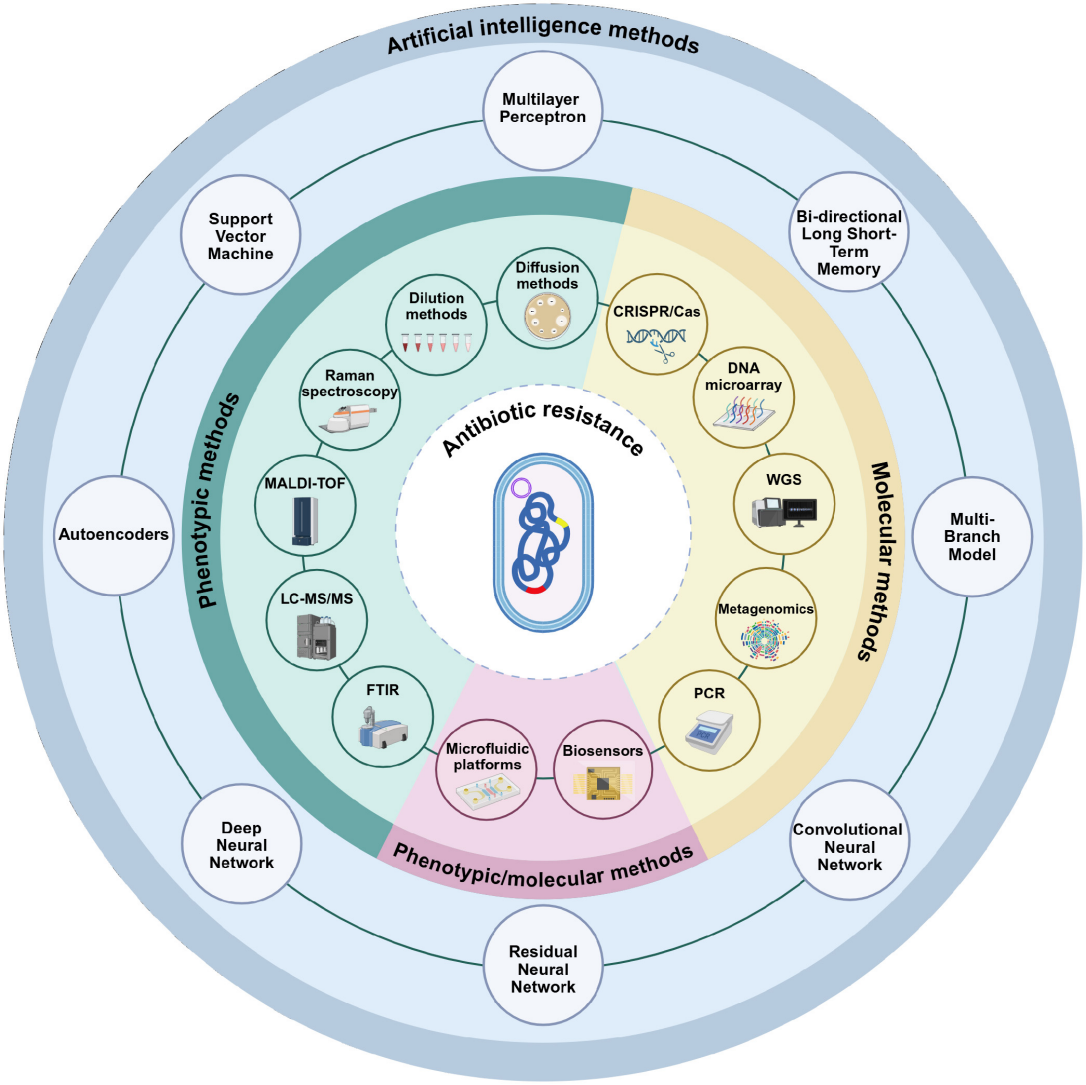
Phenotypic, molecular, and artificial intelligence-based methods used in the detection of antibiotic resistance. Phenotypic assays include conventional techniques such as disk diffusion and dilution-based methods, as well as advanced analytical platforms including Raman spectroscopy, Matrix-Assisted Laser Desorption/Ionization Time-of-Flight (MALDI-TOF) Mass Spectrometry, Liquid Chromatography with tandem mass spectrometry (LC-MS/MS), and Fourier Transform Infrared Spectroscopy (FTIR). Molecular methods encompass Polymerase Chain Reaction (PCR)-based approaches, metagenomics, whole-genome sequencing (WGS), DNA microarrays, and CRISPR/Cas technologies. Microfluidic platforms and biosensors represent versatile approaches that can be applied in both phenotypic and molecular contexts. The outermost circle illustrates artificial intelligence models (including multilayer perceptrons, bi-directional long short-term memory, multi-branch architectures, convolutional neural networks, residual neural networks, deep neural networks, autoencoders, and support vector machines). Unlike phenotypic and molecular methods, these approaches are not stand-alone diagnostic tools but serve as computational frameworks that integrate with and enhance conventional methods. Their role is to support data interpretation, increase accuracy, and enable automation. Created in BioRender. Aldea, A. (2025, https://BioRender.com/mgjtdea).

Given this background, effective detection and monitoring are crucial. In this review, we provide a broad, narrative synthesis of methods for detecting antibiotic resistance, from classical culture-based approaches to advanced molecular and computational techniques (Figure 1). Several reviews have addressed different aspects of resistance detection, including molecular techniques, agroecosystem surveillance, and rapid point-of-care assays (Elbehiry et al., 2025; Yamin et al., 2023; Kaprou et al., 2021; Dietvorst et al., 2020; Anjum et al., 2018; March-Rosselló, 2017; Luby et al., 2016; McLain et al., 2016; Aarts et al., 2005; Sundsfjord et al., 2004; Tan, 2003). Building on these contributions, the present review provides an integrated perspective on phenotypic, molecular, spectroscopic, biosensing, microfluidic, and AI-enhanced approaches. We emphasize diagnostic performance, workflow considerations, and feasibility in resource-limited settings, supported by a comparative synthesis of sensitivity, specificity, turnaround time, and costs, along with a curated list of validated primers for high-priority resistance genes. Taken together, this framework is intended to guide both research and clinical applications by clarifying the comparative strengths, limitations, and future potential of current detection strategies.

## 2 Phenotypic methods for antibiotic susceptibility testing

### 2.1 Traditional phenotypic methods for antibiotic susceptibility testing

Phenotypic antimicrobial susceptibility testing (AST) remains a cornerstone in clinical microbiology, providing direct insights into bacterial responses to antibiotics. Despite the emergence of molecular and rapid diagnostic tools, diffusion and dilution methods continue to be widely employed due to their accessibility, cost-effectiveness, and ability to provide actionable clinical data. These techniques, standardized by organizations such as the Clinical and Laboratory Standards Institute (CLSI) and the European Committee on Antimicrobial Susceptibility Testing (EUCAST), allow for robust resistance surveillance and therapeutic decision-making.

From an equipment perspective, traditional phenotypic AST requires relatively basic laboratory infrastructure (e.g., laminar flow hood, autoclave, incubator). Recent market estimates indicate that laminar flow hoods cost between $6,000–$15,000 (Excedr, 2024). Microbiological incubators are comparatively less expensive, typically ranging from $1,000 to $2,500 depending on size and manufacturer (Labster, 2025). Autoclaves and sterilization systems fall within the range of $5,000–$20,000 for standard models (LabX.com, 2025c). In addition, biosafety cabinets (Class II), often required in clinical microbiology settings, are priced between $10,000 and $20,000 depending on brand and features (Excedr, 2024). Although the initial capital investment is substantial, once infrastructure is established, diffusion- and dilution-based methods remain among the most economical AST approaches.

#### 2.1.1 Diffusion methods

Diffusion-based techniques, including the disk diffusion method (Kirby-Bauer test) and the gradient diffusion method (E-test), assess bacterial susceptibility by measuring inhibition zones formed as antibiotics diffuse through agar media.

The Kirby-Bauer disk diffusion test is a standardized method: following inoculation of a bacterial suspension onto agar, antibiotic-impregnated disks are placed, incubated, and inhibition zones are measured according to CLSI/EUCAST to classify isolates as susceptible, intermediate, or resistant (Dopcea et al., 2020; Wayne, 2025; Bauer et al., 1966). This method is widely used for monitoring resistance trends due to its reproducibility, low cost, and simple standardization (Hudzicki, 2009).

However, its primary limitation is the inability to provide minimum inhibitory concentration (MIC) values (Gajic et al., 2022), restricting its utility when precise dosing is needed. The method also requires 18–24 h incubation, which may delay therapeutic decision-making, especially in severe infections requiring rapid de-escalation (Khan et al., 2019). Despite this, it remains an invaluable tool for routine susceptibility testing of major pathogens, including ESKAPE (Yin et al., 2021; Zhang et al., 2021a; Yang et al., 2019; Mendiratta et al., 2008; Cauwelier et al., 2004). Comparative evaluations have demonstrated very high diagnostic performance of disk diffusion methods, with sensitivity and specificity values frequently exceeding 95%. For example, cefoxitin disk diffusion testing for methicillin resistance in staphylococci achieved 98%–100% sensitivity and 96%–100% specificity across multicenter trials (Broekema et al., 2009; Swenson et al., 2005). Similarly, in *Staphylococcus epidermidis*, cefoxitin disk diffusion and broth microdilution showed categorical agreement values of 96%–98%, with low rates of very major and major errors, supporting their reliability for detecting *mec*A-mediated resistance (Naccache et al., 2019).

From a cost perspective, disk diffusion is highly economical, with material costs of approximately $2–$5 per test (Alizade et al., 2016). In blood-culture extended-spectrum β-lactamase (ESBL) workflows, a rapid direct test was estimated at $1.54 per test, screening/confirmatory disk diffusion assay at $2.32 per test, whereas a combined MIC screening plus ESBL E-test protocol cost $49.65 per test (Cuellar-Rodŕıguez et al., 2009). Such low per-sample costs explain its widespread use in both high- and low-resource settings.

Unlike the Kirby-Bauer test, which provides qualitative or semi-quantitative data, the E-test is a quantitative method that determines the MIC of an antibiotic (Pfaller et al., 2010; Brown and Brown, 1991). A strip with a gradient of antibiotic concentrations is placed on agar, forming an inhibition ellipse; the MIC is read at the intersection (Liu et al., 2014). The E-test offers superior precision compared to disk diffusion, making it particularly useful for MDR infections or when using antibiotics with narrow therapeutic windows (Liu et al., 2014). However, it is more expensive than disk diffusion (E-test strips cost approximately $2–$3 each) and less scalable for high-throughput testing (Reller et al., 2009). Despite these limitations, the E-test remains an essential tool for resistance surveillance and clinical decision-making. Swenson et al. (2005) confirmed its strong agreement with reference methods, reporting sensitivities and specificities above 95% when compared to broth microdilution.

#### 2.1.2 Dilution-based methods

Dilution techniques, including agar and broth dilution methods, offer precise MIC determinations and are considered the gold standard for AST. They are particularly useful for slow-growing or fastidious bacteria and for evaluating new antimicrobials in research.

Agar dilution is a quantitative reference method, involving the incorporation of serial antibiotic concentrations into agar media. Multiple bacterial isolates are spot-inoculated onto each plate, and the MIC is determined as the lowest concentration that fully inhibits visible growth after incubation (Wayne, 2025). This technique allows simultaneous testing of multiple isolates, making it suitable for epidemiological surveillance and antibiotic development studies. Comparative studies show strong correlation with gradient methods such as the E-test, supporting its reliability (Valdivieso-García et al., 2009; Glupczynski et al., 2002; Baker et al., 1991). However, its labor-intensive nature and the need for multiple agar plates per antibiotic limit its routine clinical use.

The broth dilution method is a quantitative and highly standardized approach It can be performed as macrodilution or, more commonly, broth microdilution in 96-well plates, offering greater scalability for high-throughput workflows (Wayne, 2025). Following inoculation, plates are incubated, and the MIC is determined as the lowest antibiotic concentration that prevents visible bacterial growth. Broth microdilution is the reference method for susceptibility testing of diverse pathogens, including both fast-growing and slow-growing species, as well as anaerobes (Cordovana and Ambretti, 2020; Klare et al., 2005) and certain fungi (Pfaller et al., 2010; Fleck et al., 2007). It also demonstrates high concordance with agar dilution and E-test (Wu et al., 2015; Pfaller et al., 2010; Baker et al., 1991). In recent comparative evaluations, broth microdilution was confirmed as the most reliable reference method, showing sensitivity and specificity values above 97%, and serving as the gold standard in colistin resistance testing (Chauhan et al., 2022).

### 2.2 Modern phenotypic methods for antibiotic susceptibility testing

Since traditional ASTs can take up to 72 h to provide results (Weis et al., 2022), emerging phenotypic and spectroscopic approaches aim to deliver faster susceptibility estimates using growth surrogates or biochemical fingerprints. These remain investigational, lacking standardized protocols and breakpoints, and are usually benchmarked against CLSI/EUCAST methods.

#### 2.2.1 Raman spectroscopy

Raman spectroscopy offers a label-free, non-destructive strategy for the rapid phenotypic detection of antibiotic resistance, based on the analysis of bacterial biochemical fingerprints. By illuminating bacterial samples with a laser and detecting inelastically scattered photons, characteristic Raman shifts are recorded that reflect the molecular composition of the cell (Novikov et al., 2022; Galvan and Yu, 2018). Each bacterial species can produce unique Raman spectral patterns, reflecting its composition of proteins, nucleic acids, lipids, and metabolites (Novikov et al., 2022). Both conventional Raman approaches (Verma et al., 2021) and surface-enhanced Raman spectroscopy (SERS) (Ciloglu et al., 2021) have been applied to distinguish resistant from susceptible strains based on subtle chemical differences.

Because spontaneous Raman scattering is weak, signal amplification strategies are required. SERS, for example, uses metallic nanoparticles, typically silver or gold, that localize to bacterial surfaces and amplify spectral signals from biomolecules associated with resistance phenotypes (Ardelean et al., 2022; Kearns et al., 2017). Resonance Raman spectroscopy can further boost vibrational modes by aligning excitation wavelengths with bacterial chromophores (Novikov et al., 2022).

Raman-based assays can also monitor antibiotic-induced biochemical shifts, enabling rapid AST. Following antibiotic exposure, susceptible cells show metabolic suppression, while resistant bacteria maintain their biochemical profiles (Han et al., 2020; Liu C. Y. et al., 2016). Although spectral differences can be subtle, multivariate statistical tools and machine learning (ML) algorithms have been employed for accurate classification (Ogunlade et al., 2024; Novikov et al., 2022; Ciloglu et al., 2021). SERS-based AST has successfully captured metabolic signatures correlated with MIC values, often delivering results faster than conventional methods (Liu C. Y. et al., 2016). In practice, isolates are prepared as suspensions or mixed with nanoparticles (for SERS), spectra are acquired within ~1–2 min, preprocessed (background subtraction, noise reduction, normalization), and analyzed with ML models, yielding predictions in < 30 s per sample when benchmarked against conventional AST (Lu et al., 2023; Nakar et al., 2022; Ciloglu et al., 2021).

Recent studies support the clinical utility of Raman-based platforms. In one example, multi-resistant *Escherichia coli* strains, harboring extended-spectrum β-lactamase and carbapenemase genes, were distinguished from sensitive isolates using a dual Raman strategy. UV resonance Raman spectroscopy (UVRR) enhanced nucleic acid and aromatic amino acid signals, revealing a higher nucleic acid-to-protein ratio in resistant strains. Complementary Raman microspectroscopy captured single-cell spectral features. ML models trained on these data achieved accurate classification, with spectral variation reflecting both qualitative and quantitative differences in genomic content due to the presence of multiple resistance determinants (Nakar et al., 2022).

SERS coupled with deep neural networks (DNNs) has also shown strong performance. In one study (Ciloglu et al., 2021), MRSA and Methicillin-Sensitive *S. aureus* (MSSA) were distinguished using SERS spectra acquired with silver nanoparticle (AgNPs) substrates, capturing subtle differences in the chemical composition of the bacterial cell walls. This occurs due to the strong influence of the cell wall components on the SERS spectral features, as silver nanoparticles tend to aggregate on the cell surface, enhancing the Raman signal from this region (Efrima and Zeiri, 2009). A stacked autoencoder-based model trained on raw spectral data achieved high classification accuracy between MRSA and MSSA. Specifically, the SAE-based deep learning model reached 97.66% accuracy and an Area Under the Curve (AUC) of 0.99 in distinguishing MRSA from MSSA (Ciloglu et al., 2021).

A novel Raman-based AST method utilizes deuterium incorporation from heavy water (D_2_O) to track bacterial metabolism. Live bacteria incorporate deuterium into C-D bonds, producing distinct spectral peaks in the “silent” region (~2,040–2,300 cm^−1^) where there is little interference (Xu et al., 2017). In the presence of an effective antibiotic, susceptible bacteria's metabolism slows dramatically, leading to a much weaker C-D Raman signal, whereas resistant bacteria continue to grow and incorporate D, yielding a strong C-D peak (Single Cell Biotech, 2025). Using stimulated Raman scattering microscopy, susceptibility profiles were generated within 2.5 h, with over 98% classification accuracy for *Mycobacterium tuberculosis*, including from direct sputum samples (Ogunlade et al., 2024).

Further validation has been reported across different pathogens. Spencer et al. (2011) showed that Raman spectroscopy identified MRSA vs. MSSA with 90.2% accuracy (sensitivity 96%, specificity 85%), and distinguished MRSA with reduced susceptibility to vancomycin from standard MRSA with 96.3% accuracy (sensitivity 100%, specificity 93%). Similarly, Lu et al. (2023) demonstrated that a random forest classifier applied to single-cell Raman spectra distinguished carbapenem-resistant *A. baumannii* with 99.92 ± 0.06% accuracy, supported by Receiver Operating Characteristic (ROC) analysis with an AUC of 1.0, indicating near-perfect sensitivity and specificity. Reported limits of detection (LoD) range from 10^3^ CFU/ml to as low as 10–15 CFU/ml, depending on the specific platform and detection strategy (Chang et al., 2019; Wang K. et al., 2018).

Raman instruments range from portable units at $10,000–$50,000 to benchtop systems ($20,000–$200,000) and high-end confocal/multi-laser platforms exceeding $400,000 (Barnett Technical Services, 2025; Excedr, 2025b; Henderson, 2024). Additionally, a low-cost ($5,000) portable Raman microscope was developed for low-resource settings (Ogunlade et al., 2024). Consumables vary: commercial SERS substrates cost < $2–$25 per test (some >$100) (Thermo Scientific ProGolab, 2010), while low-cost research substrates can be fabricated for $1.20 per substrate (Yu et al., 2019) or even ~$0.10 per mm^2^ (Kesava Rao et al., 2024). Conventional Raman substrates (quartz, CaF_2_ slides) cost ~$75–$230 per unit (Corporation, 2025; Ltd, 2025a,b), but are reusable.

#### 2.2.2 Matrix-assisted laser desorption/ionization time-of-flight (MALDI-TOF) mass spectrometry

Matrix-assisted laser desorption/ionization time-of-flight (MALDI-TOF) mass spectrometry (MS) has advanced pathogen identification since 1990s (Claydon et al., 1996; Holland et al., 1996) and accelerated antimicrobial resistance (AMR) detection. It works by mixing a sample, such as a bacterial colony, with a matrix compound and using laser ionization to generate charged protein fragments. These ions travel through a time-of-flight tube, producing a unique mass spectrum or “fingerprint” of the organism (Florio et al., 2020). For AMR testing, workflows typically expose standardized inocula to antibiotics for 90 min–5 h, then acquire paired spectra (with/without drug) using matrix-assisted spotting. Susceptibility can be inferred from growth ratios or entire spectra analyzed with ML (Lin et al., 2025; Ren et al., 2024; Axelsson et al., 2020; Idelevich et al., 2018). Several approaches have been developed, including bacterial growth detection after antibiotic exposure (Idelevich et al., 2018), identification of resistance-associated mass spectral profiles (Weis et al., 2022), analysis of antibiotic modifications due to bacterial enzymatic activity (Hrabák et al., 2013), and analysis of the proteomic changes induced by the antibiotic exposure stress (Haider et al., 2025). Compared to traditional antibiotic susceptibility tests and DNA amplification, MALDI-TOF delivers faster results, often within minutes once a colony is obtained (Kostrzewa et al., 2013).

Beyond its accuracy in species identification (Cassagne et al., 2016; De Bruyne et al., 2011), MALDI-TOF has shown strong performance in AMR detection, achieving near-perfect accuracy in some contexts. For example, β-lactamase-mediated hydrolysis assays reached 98% sensitivity and 100% specificity after 30 min of incubation and 100% for both at 60 min. Direct-on-target microdroplet growth assay (DOT-MGA) identified meropenem resistance with 100% sensitivity and specificity in *K. pneumoniae* and slightly lower in *P. aeruginosa*. Biomarker-based assays show variable performance: 96% sensitivity and 73% specificity for an *Acinetobacter*-derived cephalosporinase (ADC), or ~100% specificity of the phenol-soluble modulin (PSM)-mec peptide for MRSA detection (Florio et al., 2020). Validation across clinical samples is strong: MBT-ASTRA achieved 99% sensitivity/specificity and 97% accuracy on 841 blood cultures (Axelsson et al., 2020), while DOT-MGA confirmed 100% accuracy for *K. pneumoniae* (after 4 h) and *P. aeruginosa* (after 5 h) (Idelevich et al., 2018). ML applications have also proven promising, with accuracies of 67%–97% in *E. coli* isolates (Lin et al., 2025) and Area Under the Receiver Operating Characteristic Curve (AUROC) values ranging from 0.80 to 0.95 in >1,000 *S. epidermidis* isolates (Ren et al., 2024). Reported LoD typically range from ~10^5^ CFU/ml down to ~10^3^ CFU/ml when optimized workflows such as membrane filtration are used, with most hydrolysis assays requiring standardized inocula of ~10^7^–10^8^ CFU/ml for reliable detection (Haider et al., 2023; Ghebremedhin et al., 2016; Hrabák, 2014; Papagiannitsis et al., 2015). However, the sensitivity of this method for detecting resistance markers varies depending on the mechanism. While enzymatic antibiotic degradation and abundant biomarkers are easily identifiable (Hrabák et al., 2013), subtle changes such as point mutations in target enzymes may not produce distinct spectra. For example, fluoroquinolone resistance involves subtle amino acid substitutions that alter DNA gyrase or topoisomerase IV without producing a unique degradation product (Redgrave et al., 2014). Rifampin resistance also results from point mutations that change the structure of RNA polymerase but do not necessarily lead to detectable enzymatic activity changes (Goldstein, 2014).

A key limitation of the technique is that it typically requires an isolated colony to generate a high-quality spectrum, meaning a cultivation step is necessary (Idelevich et al., 2018; De Bruyne et al., 2011). Additionally, the mass spectrum can be significantly affected by an insufficient or excessive sample amount (Liu et al., 2007). Cost-wise, instruments are expensive, ranging from $200,000 to $500,000 with annual maintenance of $25,000–$30,000 (Excedr, 2025a; Tran et al., 2015). However, per-test costs are low: $0.20–$1.50 when analyzing bacterial colonies (Patel, 2013; Cherkaoui et al., 2010), and $1.5–$7 for blood cultures depending on workflow or kits used (Han et al., 2021; Zhou et al., 2017).

#### 2.2.3 Liquid chromatography-tandem mass spectrometry (LC-MS/MS)

LC-MS/MS integrates liquid chromatography (LC) for molecular separation with MS for high-resolution detection, typically employing electrospray ionization. It identifies proteins, peptides, and metabolites by ionizing analytes, separating them by mass-to-charge ratio, and fragmenting selected ions for structural resolution (Grebe and Singh, 2011). This method offers exceptional sensitivity, allowing for the identification of resistance markers and antibiotic metabolites at sub-nanomolar concentrations (Mokh et al., 2017; Wang et al., 2017). Recent studies have reported very high diagnostic performance: for instance, LC-MS/MS showed a sensitivity of 97.6%–100% and specificity of 91%–100% in detecting carbapenemase-producing *Enterobacterales* (Li G. et al., 2022), while for resistance mediated by the TetX enzyme (encoded by *tet*X gene) the method achieved 98.9% sensitivity and 100% specificity when compared to PCR (Zhang L. et al., 2024). Reported LoD range from ~10^7^ CFU/ml in hydrolysis assays to ~10^3^ CFU/ml in optimized targeted workflows (Foudraine et al., 2022; Peaper et al., 2013).

LC-MS/MS has been applied in AMR research to detect key resistance determinants in bacterial pathogens. For instance, a proof-of-concept study demonstrated the ability of high-resolution LC-MS/MS to identify four major carbapenemase enzymes (KPC, NDM, VIM, and OXA-48) in *E. coli* and *K. pneumoniae* isolates (Foudraine et al., 2019). More recently, LC-MS/MS was used in a targeted proteomics approach (Foudraine et al., 2022) to detect resistance markers in *E. coli* and *K. pneumoniae* from positive blood cultures. This method enabled the rapid identification of β-lactamases (e.g., SHV, CTX-M, KPC, NDM), aminoglycoside-modifying enzymes, 16S rRNA methyltransferases, and quinolone resistance mutations. Protein digestion and peptide profiling yielded resistance signatures within ~3 h, considerably shortening turnaround time. Workflows generally involve either short incubations (1–2.5 h) with antibiotics to detect enzymatic degradation products, or protein extraction and tryptic digestion ( 3 h including LC-MS/MS run) for peptide analysis. Extracts are separated on C18 columns and analyzed by MS/MS, with resistance signatures identified through targeted transitions or multiplex peptide profiling. This modular design supports both focused 1-h assays (e.g., carbapenemase, *tet*X) and broader multiplex panels covering dozens of determinants (Zhang L. et al., 2024; Foudraine et al., 2022; Li G. et al., 2022; Foudraine et al., 2019).

Beyond resistance detection, LC-MS/MS is widely used for antibiotic monitoring in clinical and environmental contexts. It detects antibiotics and metabolites in complex samples, aiding studies on degradation and resistance mechanisms (Yipel et al., 2017; Blair et al., 2015; Fedorova et al., 2014). Instruments typically cost $75,000–$500,000, depending on configuration and whether new or refurbished (MarketsandMarkets, 2024).

#### 2.2.4 Fourier-transform infrared (FTIR) spectroscopy

Fourier-transform infrared (FTIR) spectroscopy has emerged as a promising phenotypic tool for the rapid detection of antibiotic resistance, leveraging biochemical alterations that accompany resistance development. FTIR spectra reflect the molecular composition of bacterial cells, capturing absorption peaks from proteins, lipids, nucleic acids, and carbohydrates (Beć et al., 2020). Because resistance often alters cell wall structure, enzyme production, or lipid composition (Blair et al., 2015; Lin et al., 2015; Garcia-Bustos and Tomasz, 1990), FTIR can detect these changes in characteristic vibrational bands. Relevant regions include proteins (1,500–1,800 cm^−1^) (Kariakin et al., 2002), carbohydrates (900–1,200 cm^−1^) (Naumann, 2001), and fatty acids (2,800–3,100 cm^−1^) (Shapaval et al., 2019).

Sample preparation is minimal: a dried film or bacterial pellet is applied to an IR-transparent slide (e.g., ZnSe), and spectra are collected in the 4,000–600 cm^−1^ range within minutes (Maity et al., 2013). Typically, cells are concentrated, spotted on ZnSe slides, air-dried, scanned (128 scans, 4 cm^−1^), preprocessed (baseline correction, normalization), and analyzed with ML algorithms, enabling results in 20–40 min (Abu-Aqil et al., 2024; Suleiman et al., 2022).

When paired with ML, FTIR can significantly enhance diagnostic performance. For example, susceptibility of *P. aeruginosa* was predicted in < 20 min with 82%–90% accuracy, 81%–92% sensitivity, and 66%–79% specificity (Suleiman et al., 2022). Similarly, *E. coli* strains were classified as resistant or susceptible with ~85% accuracy following 24-h incubation (Sharaha et al., 2017). In a larger cohort, ESBL-positive *E. coli* were detected with 97%–99% sensitivity, 94% specificity, and 98% overall accuracy (Sharaha et al., 2019). A 2024 study on *K. pneumoniae* analyzed >27,000 spectra from 636 isolates, reporting >95% accuracy in strain identification and 74%–81% sensitivity in resistance classification (Abu-Aqil et al., 2024). Suleiman et al. (2021) further showed that FTIR microspectroscopy enabled the detection of ESBL-producing *K. pneumoniae* with ~89% accuracy, ~88% sensitivity, and ~89% specificity within 20 min after culture. In addition, Wijesinghe et al. (2021) demonstrated that a portable attenuated total reflectance (ATR)-FTIR system could classify ceftriaxone-resistant *E. coli* harboring the *bla*_CTX-M_ gene with 89.2% sensitivity and 66.7% specificity, suggesting the feasibility of low-cost clinical deployment. Reported LoD range from 10^3^ to 10^5^ CFU/ml, with one recent study demonstrating detection at ~10^4^ CFU/ml in complex wound samples (Chen et al., 2022).

FTIR has also been applied in outbreak surveillance. In one multicenter evaluation, the IR spectral clustering of clinical isolates closely mirrored genotyping-based groupings, enabling early recognition of epidemic strains. The technology has been used to identify ESBL-producing *K. pneumoniae* and to build a national spectral database in Israel, which subsequently facilitated the detection of novel carbapenem-resistant clones (Lurie-Weinberger et al., 2025).

Overall, FTIR offers a reagent-free, non-destructive platform for detecting resistance (Salman et al., 2017). It supports early phenotype identification, integrates into clinical workflows, and is applicable to many pathogens. However, it requires prior culturing, limiting direct-from-sample use (Abu-Aqil et al., 2024). Spectral reproducibility is highly dependent on the standardization of sample preparation and growth conditions, and spectral interpretation requires advanced computational tools (Abu-Aqil et al., 2024; Salman et al., 2017). Moreover, spectral shifts may be non-specific, reflecting general physiological or metabolic changes rather than directly indicating resistance mechanisms (Jin et al., 2017). Instrumentation costs and the need for technical expertise can also be barriers in resource-limited settings (Suleiman et al., 2022).

Economically, FTIR systems cost $15,000–$100,000 (high-end up to $150,000) (LabX.com, 2025g). Attenuated Total Reflectance (ATR)-FTIR has negligible consumables, while transmission mode using KBr pellets adds ~$0.7/sample (International Crystal Laboratories, 2025; Shepel et al., 2015) and polytetrafluoroethylene infrared (PTFE IR) cards ~$4 per sample (International Crystal Laboratories, 2024), with costs depending on substrate reuse policies.

## 3 Molecular methods to detect the antibiotic resistance genes

Various methods have been developed over time to detect antibiotic resistance genes (ARGs) in environmental or biological samples. These include techniques like polymerase chain reaction (PCR), quantitative PCR (qPCR), and digital PCR (dPCR) using specific primers targeting ARGs; WGS; DNA microarray technology; metagenomics; and the application of the CRISPR/Cas system. Molecular approaches provide high sensitivity, specificity, and rapid turnaround times, making them indispensable in clinical and environmental surveillance of AMR.

### 3.1 PCR, qPCR and dPCR

PCR, invented in 1983 by Kary Mullis (Mullis et al., 1986) amplifies specific DNA fragments through repeated cycles of denaturation, annealing, and extension (Al-Zaidi et al., 2022). qPCR (real-time PCR) enables DNA quantification using fluorescent dyes (Heid et al., 1996), while dPCR partitions samples into thousands of reactions, allowing absolute quantification without standard curves (Vogelstein and Kinzler, 1999). Both significantly improved ARG detection by increasing sensitivity and precision. DNA extraction remains a critical step before amplification, requiring optimized kits to minimize inhibitors.

Platform costs vary: conventional PCR machines cost $1,500–$50,000 ($750–$25,000 for second hand), qPCR systems $8,000–$100,000 ($2,500–$90,000 for second hand) (LabX.com, 2025e), and dPCR units cost $50,000–$200,000 ($20,000–$100,000 for second hand) (LabX.com, 2025f). Per-test costs range from $0.22 to $10, depending on method and kit (Applied Biological Materials, 2025; Lab Manager, 2025; MilliporeSigma, 2025; Roberts, 2014).

While conventional PCR remains widely used, its qualitative nature limits gene abundance analysis (Lin and Di, 2020). qPCR improves upon this by enabling real-time quantification, offering greater sensitivity and precision (Heid et al., 1996). dPCR advances this approach by allowing absolute quantification without the need for a standard curve (Gobbo et al., 2024), which is particularly advantageous for detecting low-abundance ARGs in challenging matrices such as wastewater (Ferraro et al., 2024; Maestre-Carballa et al., 2024; Singh et al., 2024) and soil (Griffin et al., 2019; Cavé et al., 2016). For example, Maestre-Carballa et al. (2024) applied dPCR in a city-wide monitoring framework to quantify *sul*2 and *tet*W genes in hospital wastewater and seawater, reporting absolute abundances of 6,000–18,600 copies/ng DNA, while metagenomics provided broader resistome coverage but with lower sensitivity.

Clinical evaluations show variable performance. A one-step digital droplet PCR platform applied directly to whole blood achieved 100% sensitivity and 100% specificity for *bla*_CTX-M_, *bla*_*KPC*_, *bla*_*OXA*−48_, *mec*A, and *van*A (Abram et al., 2020). A multiplex qPCR assay reached 97.44% sensitivity and 96.15% specificity for *mec*A detection in clinical *S. aureus* isolates, with an AUC of 0.98 for MRSA diagnosis (Lee et al., 2024). In contrast, multiplex PCR on orthopedic infection samples showed lower sensitivity (46%) but high specificity (95%), varying by pathogen-antibiotic combination (e.g., 100% sensitivity for oxacillin resistance in *S. aureus*, but 33% sensitivity for aminoglycoside resistance in enterococci) (Sigmund et al., 2020). Beyond these, Abram et al. (2020) developed a culture-free blood dPCR platform able to detect resistant bacteria at 10 CFU/ml within 1 h, with 100% sensitivity and specificity for key ARGs such as *bla*_CTX-M_, *bla*_KPC_, *bla*_OXA-48_, *mec*A, and *van*A. Reported LoD vary across PCR platforms, typically ~10^2^–10^4^ genome copies for conventional PCR, ~10–100 genome copies per reaction for qPCR, and as low as 1–2 copies per reaction for dPCR (Keenum et al., 2022; Cavé et al., 2016; Chandrashekhar et al., 2015; Böckelmann et al., 2009).

Multiplex PCR has enhanced ARG detection by enabling simultaneous amplification of multiple genes (Wang et al., 2021; Strommenger et al., 2003). Integration with metagenomics expands resistome coverage (Sukhum et al., 2019), while combining PCR with sequencing supports comprehensive resistome analysis. Furthermore, high-throughput qPCR (HT-qPCR) allows parallel detection of hundreds of ARGs with LoD as low as 10^–4^ ARGs per 16S rRNA gene, and has been applied globally in soils, wastewater, and gut microbiomes (Waseem et al., 2019).

As a resource for the scientific community, we assembled in Table 1 a consolidated set of validated primer sequences for the most frequently reported ARGs (Zhuang et al., 2021). These genes were selected based on their high prevalence, and the primer pairs were taken from the original design publications, prioritizing those most widely adopted in subsequent studies. By integrating scattered information from diverse studies into a single curated reference, this table is intended to facilitate assay design and promote standardized approaches to ARG detection across clinical, environmental, and research contexts.

**Table 1:** Primer pairs selected for the detection of ARGs reported in the analyzed studies.

**Gene family^a^**	**Gene**	**Primer sequence**	**Amplicon size (bp)**	**Annealing T°C**	**References**
AG-R	*aad*A	5′-TGATTTGCTGGTTACGGTGAC-3′	284	58°C	Van et al. (2008)
		5′-CGCTATGTTCTCTTGCTTTTG-3′			
	*arm*A	5′-CCGAAATGACAGTTCCTATC-3′	774	55°C	Yan et al. (2004)
		5′-GAAAATGAGTGCCTTGGAGG-3′			
	*rmt*B	5′-ATGAACATCAACGATGCCCT-3′	769	55°C	Yan et al. (2004)
		5′-CCTTCTGATTGGCTTATCCA-3′			
BL-R	*bla*_CTX-M_ group 1^b, c^	5′-GACGATGTCACTGGCTGAGC-3′	499	55°C	Pitout et al. (2004)
		5′-AGCCGCCGACGCTAATACA-3′			
	*bla*_CTX-M_ group 2^b, c^	5′-GCGACCTGGTTAACTACAATCC-3′	351	55°C	Pitout et al. (2004)
		5′-CGGTAGTATTGCCCTTAAGCC-3′			
	*bla*_CTX-M_ group 3^b, c^	5′-CGCTTTGCCATGTGCAGCACC-3′	307	55°C	Pitout et al. (2004)
		5′-GCTCAGTACGATCGAGCC-3′			
	*bla*_CTX-M_ group 4^b, c^	5′-GCTGGAGAAAAGCAGCGGAG-3′	474	62°C	Pitout et al. (2004)
		5′-GCTCAGTACGATCGAGCC-3′			
	*bla*_IMP-1_	5′-ATGAGCAAGTTATCTGTATTCT-3′	741	50°C	Zarrilli et al. (2004)
		5′-TTAGTTGCTTGGTTTTGATGG-3′			
	*bla*_KPC_^c^	5′-ATGTCACTGTATCGCCGTCT-3′	892	55°C	Ribeiro et al. (2016); Bradford et al. (2004)
		5′-TTTTCAGAGCCTTACTGCCC-3′			
	*bla*_NDM_^c^	5′-AAATGGAAACTGGCGACC-3′	439	52°C	Mlynarcik et al. (2016)
		5′-TAAAATACCTTGAGCGGGC-3′			
	*bla*_OXA-1_	5′-ATATCTCTACTGTTGCATCTCC-3′	619	54°C	Colom et al. (2003)
		5′-AAACCCTTCAAACCATCC-3′			
	*bla*_OXA-23_	5′-AAGCATGATGAGCGCAAAG-3′	1066	50°C	Donald et al. (2000); Senkyrikova et al. (2013)
		5′-AAAAGGCCCATTTATCTCAAA-3′			
	*bla*_OXA-48_	5′-GCTTGATCGCCCTCGATT-3′	281	57°C	Dallenne et al. (2010)
		5′-GATTTGCTCCGTGGCCGAAA-3′			
	*bla*_OXA-58_	5′-GTTGTATGTAGAGCGCAGAGG-3′	91	60°C	Mentasti et al. (2020)
		5′-ACCCACATACCAACCCACTTG-3′			
	*bla*_SHV_^c^	5′-TCGCCTGTGTATTATCTCCC-3′	768	50°C	Maynard et al. (2003)
		5′-CGCAGATAAATCACCACAATG-3′			
	*bla*_TEM-1_	5′-CATTTTCGTGTCGCCCTTATTC-3′	800	60°C	Dallenne et al. (2010)
		5′-CGTTCATCCATAGTTGCCTGAC-3′			
	*bla*_VIM-1_^c^	5′-TTATGGAGCAGCAACGATGT-3′	920	55°C	Yan et al. (2001)
		5′-CAAAAGTCCCGCTCCAACGA-3′			
	*bla*_VIM-2_^c^	5′-AAAGTTATGCCGCACTCACC-3′	865	55°C	Yan et al. (2001)
		5′-TGCAACTTCATGTTATGCCG-3′			
CN-R	*mcr*-1	5′-CGGTCAGTCCGTTTGTTC-3′	334	54°C	Liu Y.-Y. et al. (2016); Cao et al. (2020)
		5′-CTTGGTCGGTCTGTAGGG-3′			
GP-R	*van*A	5′-GGGAAAACGACAATTGC-3′	732	54°C	Dutka-Malen et al. (1995)
		5′-GTACAATGCGGCCGTTA-3′			
	*van*B	5′-ATGGGAAGCCGATAGTC-3′	635	54°C	Dutka-Malen et al. (1995)
		5′-GATTTCGTTCCTCGACC-3′			
MC-R	*ere*A	5′-AACACCCTGAACCCAAGGGACG-3′	420	52°C	Sutcliffe et al. (1996)
		5′-CTTCACATCCGGATTCGCTCGA-3′			
	*erm*A	5′-TCTAAAAAGCATGTAAAAGAA-3′	645	52°C	Sutcliffe et al. (1996)
		5′-CTTCGATAGTTTATTAATATTAGT-3′			
	*erm*B	5′-GAAAAAGTACTCAACCAAATA-3′	639	45°C	Nguyen et al. (2009)
		5′-AATTTAAGTACCGTTACT-3′			
	*erm*C	5′-TCAAAACATAATATAGATAAA-3′	642	45°C	Nguyen et al. (2009)
		5′-GCTAATATTGTTTAAATCGTCAAT-3′			
	*erm*F	5′-CGGGTCAGCACTTTACTATTG-3′	466	64°C	Chung et al. (1999)
		5′-GGACCTACCTCATAGACAAG-3′			
	*erm*G	5′-TCACATAGAAAAAATAATGAATTGCATAAG-3′	652	55°C	Patterson et al. (2007)
		5′-CGATACAAATTGTTCGAAACTAATATTGT-3′			
	*erm*Q	5′-CACCAACTGATATGTGGCTAG-3′	154	60°C	Koike et al. (2010)
		5′-CTAGGCATGGGATGGAAGTC-3′			
	*mph*E	5′-ATATGGACAAAGATAGCCCG-3′	271	68°C	Rose et al. (2012)
		5′-ATGCCCAGCATATAAATCGC-3′			
	*msr*E	5′-GCCGTAGAATATGAGCTGAT-3′	395	68°C	Rose et al. (2012)
		5′-TATAGCGACTTTAGCGCCAA-3′			
PH-R	*cml*A	5′-TGTCATTTACGGCATACTCG-3′	435	55°C	Guerra et al. (2001)
		5′-ATCAGGCATCCCATTCCCAT-3′			
	*flo*R	5′-GTCATTCCTCACCTTCATCCTAC-3′	243	60°C	Khan et al. (2011)
		5′-GACACCAGCACTGCCATTG-3′			
SF-R	*sul*1	5′-CGGCGTGGGCTACCTGAACG-3′	433	69°C	Hoa et al. (2008)
		5′-GCCGATCGCGTGAAGTTCCG-3′			
	*sul*2	5′-GCGCTCAAGGCAGATGGCATT-3′	293	69°C	Hoa et al. (2008)
		5′-GCGTTTGATACCGGCACCCGT-3′			
	*sul*3	5′-TCAAAGCAAAATGATATGAGC-3′	787	50°C	Heuer and Smalla (2007)
		5′-TTTCAAGGCATCTGATAAAGAC-3′			
TE-R	*tet*A	5′-GCTACATCCTGCTTGCCTTC-3′	211	53°C	Nawaz et al. (2006)
		5′-GCATAGATCGCCGTGAAGAG-3′			
	*tet*B	5′-CGCGGCATCGGTCATT-3′	54	50°C	Walsh et al. (2011)
		5′-GAACCACTTCACGCGTTGAGA-3′			
	*tet*C	5′-CTTGAGAGCCTTCAACCCAG-3′	418	55°C	Ng et al. (2001)
		5′-ATGGTCGTCATCTACCTGCC-3′			
	*tet*G	5′-GTCGATTACACGATTATGGC-3′	432	57°C	Yu et al. (2005)
		5′-CACTTGGCCGATCAGTTGA-3′			
	*tet*L	5′-ACTCGTAATGGTTGTAGTTGC-3′	625	58°C	Prichula et al. (2016); Frazzon et al. (2010)
		5′-TGTAACTCCGATGTTTAACACG-3′			
	*tet*M	5′-GTGGACAAAGGTACAACGAG-3′	406	55°C	Warsa et al. (1996)
		5′-CGGTAAAGTTCGTCACACAC-3′			
	*tet*O	5′-AACTTAGGCATTCTGGCTCAC-3′	515	55°C	Ng et al. (2001)
		5′-TCCCACTGTTCCATATCGTCA-3′			
	*tet*Q	5′-AGAATCTGCTGTTTGCCAGTG-3′	169	63°C	Aminov et al. (2001)
		5′-CGGAGTGTCAATGATATTGCA-3′			
	*tet*W	5′-GAGAGCCTGCTATATGCCAGC-3′	168	64°C	Aminov et al. (2001)
		5′-GGGCGTATCCACAATGTTAAC-3′			
	*tet*X	5′-CAATAATTGGTGGTGGACCC-3′	468	55°C	Ng et al. (2001)
		5′-TTCTTACCTTGGACATCCCG-3′			
MR	*cfr*	5′-TGAAGTATAAAGCAGGTTGGGAGTCA-3′	746	48°C	Kehrenberg and Schwarz (2006)
		5′-ACCATATAATTGACCACAAGCAGC-3′			
	*mec*A	5′-ATGCGCTATAGATTGAAAGGAT-3′	163	60°C	Bergeron et al. (2015)
		5′-TACGCGATATCTAACTTTCCTA-3′			

^a^AG-R, aminoglycoside resistance; BL-R, β-lactam resistance; CN-R, colistin resistance; GP-R, glycopeptide resistance; MC-R, macrolide resistance; PH-R, phenicol resistance; SF-R, sulfonamide resistance; TE-R, tetracycline resistance; MR, multiple resistance.

^b^Group 1 includes *bla*_CTX-M-1, -3, -10, -11, -12, -15, -22, -23, -28, -29, and -30_; Group 2 includes *bla*_CTX-M-2, -4, -7, and -20_; Group 3 includes *bla*_CTX-M-8_; Group 4 includes *bla*_CTX-M-9, -13, -14, -16, -17, -18, -19, -21, and -27_.

^c^To differentiate between the various gene subtypes, sequencing of the gene is required.

Despite their broad applicability, each PCR-based method has notable limitations. Conventional PCR is qualitative (Lin and Di, 2020); qPCR needs standard curves and is affected by inter-lab variability (Maestre-Carballa et al., 2024; Quthama et al., 2024; Abram et al., 2020); multiplex PCR has high specificity but variable sensitivity across pathogen-antibiotic pairs (Sigmund et al., 2020); HT-qPCR cannot optimize all primers individually and is expensive (Waseem et al., 2019); dPCR, while highly sensitive, involves high consumable costs, platform variability, and risk of false positives (Maestre-Carballa et al., 2024; Abram et al., 2020; Whale et al., 2016).

### 3.2 DNA microarray

DNA microarrays are compact analytical platforms that contain thousands of immobilized DNA probes on a solid surface. They enable high-throughput, parallel detection of specific genetic sequences through hybridization-based methods, facilitating the simultaneous interrogation of gene expression, genetic variation, or microbial identity across complex samples (Heller, 2002). In AMR research, DNA microarrays enable rapid genotypic profiling of resistance genes across bacterial isolates in a single assay (Call et al., 2003). Unlike PCR, which targets one or a few genes at a time, microarrays permit broad-spectrum detection of resistance determinants within a single assay simultaneously (Card et al., 2013; Rasooly and Herold, 2008), offering a more comprehensive assessment of resistomes.

In practice, microarray detection involves hybridizing fluorescently labeled DNA from the test organism to complementary oligonucleotide probes, each specific for a known resistance gene or variant. Post-hybridization washing and laser scanning reveal signal intensities, which are computationally analyzed to infer gene presence (Gwida et al., 2020). This enables detection of hundreds of resistance genes in one run (Fink et al., 2019; Song et al., 2019). Workflows often combine ligation-based hybridization, PCR amplification of perfectly matched products, hybridization on coded array spots, and scanner-based signal readout. Integrated controls at each stage ensure validity, and complete results are typically available within 7–8 h (Braun et al., 2024; Naas et al., 2011, 2010).

Clinical studies confirm diagnostic utility. For example, the AMR Direct Flow Chip achieved 100% sensitivity and specificity for detecting (*bla*_CTX-M_, *bla*_SHV_), carbapenemases (*bla*_*KPC*_, *bla*_NDM_, *bla*_VIM_, *bla*_OXA_), *mec*A, and *van* genes across 210 isolates (Fink et al., 2019), while Check-MDR CT103XL array showed over 95% concordance with WGS and multiplex PCR in identifying β-lactamase genes in resistant *Enterobacterales* isolates (Brazelton de Cardenas et al., 2021). Targeted arrays for carbapenemase genes also showed over 96% agreement with phenotypic assays and Sanger sequencing (Song et al., 2019). Naas et al. (2011) reported that the Check-MDR CT102 microarray achieved 100% sensitivity and 100% specificity for the detection of ESBL genes (*bla*_TEM_, *bla*_SHV_, *bla*_CTX-M_) and carbapenemase genes (*bla*_KPC_, *bla*_OXA-48_, *bla*_VIM_, *bla*_IMP_, *bla*_NDM-1_). In an earlier study, Naas et al. (2010) showed that the ESBL/KPC array reached sensitivities of 93% for *bla*_TEM_ and 94% for *bla*_KPC_, while *bla*_CTX-M_ and *bla*_SHV_ were detected with 100% sensitivity; specificity was 100% for all targets. Bogaerts et al. (2011) confirmed 100% sensitivity and specificity of the Check-MDR CT101 array for plasmid-mediated *bla*_ampC_, *bla*_KPC_, and *bla*_NDM_ across 207 clinical isolates. Card et al. (2013) evaluated an expanded array and reported over 91% correlation with resistance phenotypes, with an overall specificity above 83%. More recently, Braun et al. (2024) demonstrated that a DNA microarray for carbapenemase detection achieved 92.9% sensitivity and 87.7% specificity compared to whole-genome sequencing, and 95.6% sensitivity and 95.2% specificity when compared with phenotypic testing. Reported LoD are typically in the range of 10^1^–10^2^ DNA copies/μl, with some platforms detecting as few as ~30 copies/μl (Ma et al., 2020; Song et al., 2019).

However, arrays face key limitations: they rely on predefined probe sets, potentially missing novel determinants and yielding false negatives (Brazelton de Cardenas et al., 2021; Lu et al., 2014; Card et al., 2013); cross-hybridization may cause false positives (Card et al., 2013; Dally et al., 2013); and genotypic detection may not reflect phenotypic expression (Rebelo et al., 2022; Yee et al., 2021). Thus confirmatory phenotypic testing remains essential. Accessibility is also limited by specialized hardware, computational demands, and cost, and with the rise of cost-effective WGS, the scalability of arrays is increasingly questioned (Strauß et al., 2016).

Economically, scanners cost $20,000–$150,000 (refurbished $10,000–$75,000) (LabX.com, 2025b). Consumables are estimated at $40–$50/sample for microbiome arrays (Thissen et al., 2019), with overall assay costs reported at $150–400 per array (up to $500 for genome-wide arrays) plus ~$325 for processing (Narrandes and Xu, 2018).

### 3.3 Metagenomics

Metagenomics is the study of genetic material collected directly from environmental samples, such as soil, water, or animal gut, without the need to isolate or grow individual organisms (Hugenholtz and Tyson, 2008). Unlike PCR, which requires prior sequence knowledge, metagenomics allows untargeted detection of both known and novel ARGs, expanding our understanding of AMR dissemination. By leveraging high-throughput sequencing, it captures the total genomic content, including uncultivable microorganisms, providing a comprehensive view of microbial diversity and resistance (Handelsman, 2004).

Three complementary strategies are commonly used: amplicon sequencing (e.g. 16S/ITS/18S) for taxonomic profiling but limited ARG insights (Matchado et al., 2024); shotgun sequencing, which reconstructs community structure and detects known and novel ARGs, often linking them to mobile genetic elements (MGEs) or specific hosts (Usyk et al., 2023; Quince et al., 2017); and functional metagenomics, which bypasses sequence databases entirely by cloning environmental DNA fragments into expression vectors and selecting under antibiotic pressure. This experimental framework has proven especially powerful in uncovering novel resistance determinants that remain invisible to purely sequence-based approaches (Willms et al., 2019; Dos Santos et al., 2017).

High-quality DNA extraction is the first step and must maximize yield, particularly in low-abundance carriers of ARGs (Bag et al., 2016). Library prep, purification, and quality control typically take 3–9 h, sequencing 6–48 h depending on platform, and data analysis another 4–5 h (Campos-Madueno et al., 2024). Illumina short reads provide high accuracy (Brown et al., 2021), while long-read platforms [PacBio (Simões et al., 2016), Oxford Nanopore (Ashton et al., 2015)] reconstruct full-length genes and MGEs. The sequencing process generates millions of short DNA fragments, each representing a part of a microbial genome, which must then be assembled for analysis (Shendure et al., 2017). Once sequencing data are generated, analysis typically begins with quality control and trimming [e.g. FastQC (Leggett et al., 2013), Trimmomatic (Bolger et al., 2014), Cutadapt (Martin, 2011)], followed by assembly and binning using tools such as MEGAHIT (Li et al., 2015), metaSPAdes (Nurk et al., 2017) or MetaBAT2 (Kang et al., 2019). These workflows can also reconstruct metagenome-assembled genomes (MAGs), providing higher-resolution insights into individual community members and their associated ARGs (Parks et al., 2017). Taxonomic profiles are then inferred with classifiers like Kraken2 (Wood et al., 2019), Kaiju (Menzel et al., 2016) or MetaPhlAn 3 (Beghini et al., 2021), while ARGs are annotated with specialized pipelines including resistance gene identifier (RGI) (CARD) (Alcock et al., 2023), AMRFinderPlus (Feldgarden et al., 2019), DeepARG (Arango-Argoty et al., 2018) or ARGs-OAP (Yin et al., 2023). Increasingly, integrated platforms such as MG-RAST (Meyer et al., 2008), QIIME2 (Bolyen et al., 2019) or nf-core/mag (Krakau et al., 2022) provide streamlined, end-to-end workflows. Together, these approaches yield a comprehensive picture of resistome composition, diversity and mobility.

Sequencing platforms are costly: $50,000–$1,000,000 for new systems, $10,000–$200,000 for refurbished (LabX.com, 2025a). However, portable options such as Oxford Nanopore's MinION (~$3,000) broaden accessibility (Oxford Nanaopre Technologies, 2025), making real-time, field-deployable metagenomic sequencing accessible to smaller laboratories or resource-limited settings. Consumables remain significant, with reported costs of $130 (for multiplexed runs) to $685 (for single-sample processing) per run (Govender et al., 2021).

Metagenomics has been used to profile ARG diversity in WWTPs (Li Z. et al., 2024; Guo et al., 2017), farms (He et al., 2019; Van Gompel et al., 2019), and aquatic ecosystems (Bai et al., 2019), all major reservoirs for resistance dissemination. In clinical microbiology, it has tracked gut resistome shifts under antibiotic exposure (Xu et al., 2020) and transmission of ARGs between livestock and humans (Napit et al., 2025). Importantly, it links ARGs to MGEs such as plasmids, transposons, and integrons (Inda-Díaz et al., 2023), and reveals novel genes in hard-to-culture microbes (Suenaga, 2012).

Recent clinical studies have assessed the diagnostic accuracy of metagenomics for AMR prediction. Gan et al. (2024) reported that metagenomic next-generation sequencing (mNGS) achieved a sensitivity of 67.74% and a specificity of 85.71% for carbapenem resistance overall, with particularly high sensitivity for *A. baumannii* (94.74%). Street et al. (2022) demonstrated that nanopore metagenomic sequencing predicted 87% of resistant and 100% of susceptible phenotypes in orthopedic device infections, corresponding to a high negative predictive value. Similarly, Serpa et al. (2022) showed that for lower respiratory tract infections, metagenomics achieved a sensitivity of 70% and specificity of 95% for Gram-positive bacteria, and 100% sensitivity but lower specificity (64%) for Gram-negative bacteria. Campos-Madueno et al. (2024) further evaluated Nanopore sequencing for detection of *bla*_CTX-M_ and *bla*_DHA_ genes in stool, finding that native metagenomics had 61.1% sensitivity and 100% specificity, while a pre-enrichment approach improved sensitivity to 81.5% but reduced specificity to 75%.

Although metagenomics provides unmatched insights into the distribution and transmission of ARGs, its application is limited by high costs, computational requirements, and complex interpretation (Greninger, 2018). It may also detect DNA from non-viable organisms or contaminants (Street et al., 2022), has limited sensitivity, and turnaround times of 24–48 h (Greninger, 2018).

### 3.4 Whole genome sequencing (WGS)

WGS for antibiotic resistance detection involves determining the complete DNA sequence of a bacterial genome (Köser et al., 2014), then using bioinformatics analysis to identify genetic determinants of antibiotic resistance (Mason et al., 2018). In practice, DNA from isolates (or directly from samples in metagenomic workflows) is sequenced on high-throughput platforms (Brown et al., 2021; Simões et al., 2016; Ashton et al., 2015), and reads are mapped to reference genomes or assembled *de novo*. Resistance genes are identified through databases such as ResFinder (Bortolaia et al., 2020) or CARD (Alcock et al., 2023).

Typical workflows include DNA extraction, library prep, sequencing, quality control, and mapping or *de novo* assembly, followed by resistance gene screening and prediction of susceptibility (Ding et al., 2025; Shelburne et al., 2017; Walker et al., 2015; Tyson et al., 2015; Stoesser et al., 2013). Turnaround time depends on the platform: Illumina requires at least one day, while Oxford Nanopore can complete workflows in 7–9 h, with resistance gene detection reported in under 1 h (Ali et al., 2024; Taxt et al., 2020). Costs are also platform-dependent. For example, Mellmann et al. (2016) estimated sequencing expenses at approximately €202.49 per bacterial isolate in a hospital-based setting, whereas Bruzek et al. (2020) showed that streamlined protocols on the Illumina iSeq 100 can reduce costs to around $50–100 per sample. The investment costs for sequencing instruments themselves have been addressed earlier in this review.

WGS offers major advantages: it is untargeted and detects all resistance determinants in a genome, including novel genes, eliminating the need for multiple assays (Köser et al., 2014). Concordance with phenotypic profiles is generally high: WGS predicted 89.2% of *M. tuberculosis* phenotypes with 92.3% sensitivity and 98.4% specificity (Walker et al., 2015); achieved 87% sensitivity and 98% specificity for β-lactams in Gram-negative pathogens (Shelburne et al., 2017); and reached ~99% sensitivity and ~98% specificity for MDR *E. coli* and *K. pneumoniae* (Tyson et al., 2015; Stoesser et al., 2013). Collectively, these studies indicate sensitivities and specificities above 90%, often exceeding 95%.

In addition to detecting whether an organism is resistant, WGS can elucidate the underlying mechanisms, such as point mutations or MGEs responsible for resistance. For instance, sequencing *Helicobacter pylori* can reveal mutations in 23S rRNA or *gyr*A genes that account for clarithromycin or fluoroquinolone resistance (Fauzia et al., 2023). Moreover, WGS facilitates high-resolution phylogenetic analyses, enabling researchers to trace transmission pathways and evolutionary relationships between isolates. In the context of resistance detection, such analyses provide traceability of resistance determinants, revealing whether they arise through clonal spread, HGT, or *de novo* mutation, and showing how they disseminate in time and space. These insights support outbreak investigations and infection control measures by distinguishing between imported and locally acquired strains, as demonstrated in studies of MRSA, penicillin-resistant *Streptococcus pneumoniae*, vancomycin-resistant *Enterococcus* spp., and fluroquinolone-resistant *Clostridium difficile* (Waddington et al., 2022).

Recent applications highlight utility across contexts. In Shenzhen, WGS of 282 *M. tuberculosis* isolates showed that 80% of clusters shared identical resistance mutations, indicating clonal transmission; WGS-based susceptibility testing also outperformed conventional methods in some patients (Ding et al., 2025). In Benin, WGS of 19 ESBL-producing *E. coli* from surgical infections revealed multiple β-lactamase genes (*bla*_CTX-M-15_, *bla*_OXA-1_, *bla*_OXA-181_, *bla*_TEM-1_, and *bla*_CMY-42_), accounting for resistance to third-generation cephalosporins. Additionally, aminoglycoside resistance was linked to the presence of modifying enzyme genes such as *aph*(3”)-Ib and *aph*(6)-Id (Yehouenou et al., 2021). In agroecosystems, WGS traced 361 ARGs across poultry, farm workers, and environments, many shared via plasmids and transposons (Peng et al., 2022).

Despite its advantages, WGS faces several limitations. Low-abundance variants or genes in repetitive regions may be missed, and presence of genes does not always imply expression (Verschuuren et al., 2022; Zwe et al., 2020; Cohen et al., 2019). Its accuracy is limited by dependence on existing reference databases, so standard WGS cannot by itself detect novel or poorly characterized resistance mechanisms. Several experimental and computational strategies have been developed to address this gap. These include functional metagenomics, which enables the discovery of previously unknown resistance determinants (Zwe et al., 2020; Cohen et al., 2019; Dos Santos et al., 2017), heterologous expression screening of metagenomic libraries (Gaida et al., 2015), and transposon mutagenesis approaches such as Tn-seq or TraDIS that reveal previously unrecognized resistance loci (Fernández-Garćıa et al., 2024; Yasir et al., 2020). Genome-resolved metagenomics (MAGs, Hi-C, and single-cell sequencing) can further assign novel ARGs to their microbial hosts (McCorison et al., 2025; Kawano-Sugaya et al., 2024; Goodarzi et al., 2022). On the computational side, ML classifiers and protein structure modeling can predict resistance determinants even when sequence similarity to known genes is low (Rannon et al., 2025; Olatunji et al., 2024; Wee and Wei, 2024; Yang et al., 2023). Such strategies, however, do not overcome all limitations. Computational demands, infrastructure, and bioinformatics expertise remain barriers for clinical labs (Le et al., 2024; Waddington et al., 2022; Wyres et al., 2014). Costs and turnaround are still higher than phenotypic methods, and most workflows require prior culture (Hassall et al., 2024; Waddington et al., 2022; Ellington et al., 2017). Moreover, standardization is lacking. There are no universally accepted protocols or regulatory-approved pipelines for clinical interpretation, and discrepancies can arise between laboratories in resistance gene detection and interpretation, mainly due to differences in bioinformatic pipelines, leading to inconsistent results (Hassall et al., 2024; Verschuuren et al., 2022; Waddington et al., 2022; Ellington et al., 2017). In metagenomic applications, WGS struggles to assign resistance genes to specific pathogens within complex microbial communities, complicating clinical decision-making (Chen et al., 2025; Abramova et al., 2024).

### 3.5 CRISPR/Cas-based detection

The CRISPR/Cas system, originally identified as an adaptive immune mechanism in bacteria (Bolotin et al., 2005; Mojica et al., 2005), has been adapted into a highly sensitive and specific tool for detecting ARGs. Unlike traditional PCR-based assays, which rely on DNA amplification, CRISPR diagnostics leverage targeted enzymatic cleavage to detect ARGs directly at the genetic level (Zhang et al., 2021b). For example, Müller et al. (2016) developed a CRISPR/Cas9-based method to detect plasmid-borne ARGs by targeting and cutting plasmid DNA carrying the gene of interest. In this approach, a guide RNA (gRNA) specific to the resistance gene directs the Cas9 enzyme to cleave the plasmid, linearizing it at the gene's location. The DNA is then stained with fluorescent dyes and stretched in nanofluidic channels, where optical DNA mapping generates a unique barcode. The position of the cuts is analyzed, and if consistent breaks occur at the same location, the presence of the targeted resistance gene is confirmed.

Finding Low Abundance Sequences by Hybridization (FLASH) (Quan et al., 2019) is a CRISPR-Cas9-based diagnostic tool that enriches ARG fragments for sequencing, allowing multiplex detection of thousands of genes directly from clinical samples. FLASH has successfully identified *mec*A in MRSA and *van*A in *Enterococcus faecium*, while FLASH-TB (Tram et al., 2023) was adapted to drug-resistant *M. tuberculosis*, detecting resistance directly from sputum. Beyond Cas9-based detection, CRISPR-Cas12a has been used because of its collateral cleavage activity, which generates fluorescence upon target binding. In *A. baumannii*, Cas12a enabled rapid identification of multiple β-lactamase genes in one reaction, minimizing interference from primer dimers and offering high specificity (Wang et al., 2021). Moreover, Gong et al. (2022) reported an recombinase polymerase amplification (RPA)-Cas12a assay for *mcr*-1 with 1.6 CFU/reaction sensitivity, completing the test in < 1 h. Similarly, Li K. et al. (2024) reached 100% sensitivity/specificity for *bla*_KPC_ in 80 isolates, and Cao et al. (2023) reported 100% accuracy for *mec*A detection in 111 *S. aureus* isolates.

A recent innovation involves the Cas14VIDet system, which integrates ultrafast PCR with CRISPR/Cas14 for rapid, point-of-care detection of ARGs. Unlike Cas9 and Cas12 systems, Cas14 does not require a protospacer adjacent motif (PAM), allowing flexible target recognition and enabling the detection of single-nucleotide polymorphisms with high specificity. This method was successfully applied to identify levofloxacin resistance mutations in *H. pylori*, achieving 100% sensitivity and specificity in clinical samples, with results visible within 10 min by the naked eye (Lai et al., 2025).

Across platforms, workflows generally include nucleic acid extraction, gRNA design, and pre-amplification [PCR, RPA, or loop-mediated isothermal amplification (LAMP)]. Activated CRISPR complexes cleave labeled reporters, producing fluorescence or lateral-flow signals (Lai et al., 2025; Li K. et al., 2024; Gong et al., 2022). In contrast, Cas9-based platforms such as FLASH serve primarily as enrichment tools for next-generation sequencing panels of ARGs (Tram et al., 2023; Quan et al., 2019). Reported turnaround times ranges from < 10 min for Cas14 assays (Lai et al., 2025), ~1 h for RPA-Cas12a (Gong et al., 2022), up to 2 h for multiplex PCR-Cas12a approaches (Wang et al., 2021), whereas Cas9-based NGS workflows remain longer due to sequencing requirements (Tram et al., 2023; Quan et al., 2019).

From a practical perspective, required equipment is modest: a dry bath [~$600–$1,200 (USA Scientific, 2025b)], microcentrifuge [~$200–$800 (Laboratory Supply Network, 2025)], and micropipettes [~$1,000–$2,000 (USA Scientific, 2025a; Pipette Supplies, 2025)] (Zhou et al., 2024). A low-cost fluorescence viewer [USD $35 (miniPCR bio, 2024)] can be optionally used for endpoint readout, while a biosafety cabinet is required when handling clinical isolates. For more advanced applications, laboratories can integrate additional devices such as an isothermal fluorometer [~$5,600–$6,400 (Bimedis, 2024)] or a microplate fluorescence reader [~$10,000–$30,000 (LabX.com, 2025d)], the latter of which can be replaced by a qPCR system if already available, thereby reducing costs.

CRISPR-based diagnostics represent a highly promising approach for detecting antibiotic resistance, offering multiple advantages over conventional molecular and phenotypic methods. Technically, CRISPR systems such as Cas9, Cas12, Cas13, and Cas14 demonstrate exceptional specificity by using programmable guide RNAs to recognize and cleave resistance-associated sequences with single-nucleotide resolution, enabling detection of even subtle polymorphisms (Agha et al., 2025; Lai et al., 2025). Cas13a-based assays also achieved high accuracy: a LAMP-Cas13a assay detected OXA-48 and GES carbapenemases with 100% sensitivity/specificity at ~€10 per reaction (Ortiz-Cartagena et al., 2023), while an RPA-Cas13a assay for *bla*_KPC_ reached 96.5% sensitivity and 100% specificity in clinical isolates (Liang et al., 2023). Reported LoD range from ~10^3^–10 gene copies, depending on the specific Cas system used (Qian et al., 2023; Kaminski et al., 2021).

Compared with culture-based, PCR, or WGS methods, CRISPR offers faster turnaround times (Agha et al., 2025; Lai et al., 2025; Tram et al., 2023), making it ideal for point-of-care applications. CRISPR diagnostics are also adaptable and scalable. They can be designed to detect a wide range of resistance genes simultaneously (Quan et al., 2019) and are increasingly being integrated into portable, point-of-care platforms (Agha et al., 2025; Lai et al., 2025).

However, the limitations of CRISPR-based detection systems still restrict routine use: most assays require nucleic acid extraction and pre-amplification (Ortiz-Cartagena et al., 2023; Liang et al., 2023; Gong et al., 2022); they depend on prior sequence knowledge, limiting novel gene discovery (Quan et al., 2019); off-target or background signals can occur in complex samples (Müller et al., 2016); PAM requirements constrain Cas9/Cas12, though Cas14 overcomes this (Lai et al., 2025); and most studies validate only single genes or small cohorts, limiting scalability (Lai et al., 2025).

## 4 Advanced biosensing and nanotechnological platforms for antibiotic resistance detection

### 4.1 Microfluidic lab-on-chip platforms

Microfluidic platforms are miniaturized analytical systems that handle μl-nL volumes in microscale channels and integrate sample preparation, reaction, separation, and detection on a single chip (Wu and Mu, 2024; Haeberle and Zengerle, 2007). Rather than constituting detection methods themselves, microfluidic devices function as enabling platforms that host and accelerate established genotypic (e.g., growth monitoring, viability assays) approaches in a miniaturized and integrated format. By reducing assay volumes and providing precise control of experimental conditions, microfluidics can shorten turnaround times, improve sensitivity, and minimize reagent use (Nguyen et al., 2023). Microfluidic AST encompasses both genotypic assays, including on-chip PCR or isothermal amplification for rapid resistance gene detection, and phenotypic assays that monitor bacterial growth or viability in the presence of antibiotics within microchambers or droplets (Kaprou et al., 2021). High surface-to-volume ratios accelerate diffusion and reaction kinetics, enabling single-cell resolution and the detection of heterogeneous resistance phenotypes (Wu and Mu, 2024).

A representative example is the Light Forge platform, developed for tuberculosis drug-resistance testing. Miniaturization into nanoliter reactors reduced reagent consumption nearly 1,000-fold, and high-resolution melting analysis (HRMA) costs only about $0.30 per reaction. The device relied on low-cost components (21-MP camera, fluorescent lamp, simple thermal block, basic computer interface), making it an affordable alternative to commercial real-time PCR systems (Mbano et al., 2020). Similarly, a smartphone-based imaging flow cytometry assay for urinary tract infections eliminated fluorescence labeling and washing steps, using probe-coated microparticles and an inexpensive Complementary Metal-Oxide-Semiconductor (CMOS) phone camera with a 3D-printed dongle. The test cost just $0.26 per sample and delivered rapid, sensitive detection (Wu et al., 2018).

Several recent studies illustrate the versatility of this approach. Song et al. (2022) described a 16-channel chip with freeze-dried antibiotics pre-loaded in 15 μm chambers, enabling rapid susceptibility testing in 30 min-2 h with minimal preparation. Kandavalli et al. (2022) designed arrays of 3,000 microtraps (1.25 × 1.25 × 50 μm) that retained individual cells. Growth rates with or without antibiotics were measured in ~60 min, followed by species identification via fluorescence *in situ* hybridization (FISH) targeting 16S/23S rRNA, producing species-specific susceptibility profiles in ~2 h. Automated segmentation and growth-rate analysis were facilitated by a deep learning model (Omnipose).

Other designs emphasize throughput and MIC determination. Nguyen et al. (2023) developed a ladder-shaped microchannel chip for two-fold serial antibiotic dilutions, reducing AST turnaround from ~16–20 h to 4–5 h, with over 90% concordance to conventional methods, with a reported LoD of ~10^5^ CFU/ml when testing directly from urine. Azizi et al. (2021) introduced an egg-like multivolume microchamber (EL-MVM^2^) design, in which 10 min of diffusion from a stock solution generated a broad concentration gradient; fluorescence readouts predicted susceptibility with >97% accuracy. Their earlier N-3M nanoliter platform (Azizi et al., 2018) used resazurin reduction to report growth within 1–3 h. More recently, Wat et al. (2025) described the Self-Dilution for Faster AST (SDFAST) SlipChip, where sliding two microchips produced an antibiotic dilution series within seconds; after 4–6 h incubation, a WST-8 colorimetric assay determined MIC values, achieving ~92% agreement with reference methods for *A. baumannii, E. coli, K. pneumoniae*, and *Staphylococcus* spp.

Microfluidics also support genotypic detection. Real-time PCR chips can multiplex resistance genes, as shown by a micro/nanofluidic chip detecting carbapenemase and ESBL genes from cerebrospinal fluid within 1 h with ~94% concordance to culture (Zhang et al., 2018). The cartridge-based ePlex system identified bloodstream pathogens and *bla*_CTX-M_, *van*A, *mec*A genes with 100% accuracy (Bryant et al., 2020). Also, Wu et al. (2022) demonstrated that a microfluidic chip-based LAMP platform for carbapenemase genes achieved 97.7% sensitivity and 78.8% specificity retrospectively, and in prospective testing on blood cultures reached 100% sensitivity and 93.2% specificity, with an overall accuracy of 94%. Another LAMP device simultaneously identified *Staphylococcus* spp. (*fem*A gene) and methicillin resistance (*mec*A gene) directly from cerebrospinal fluid, distinguishing MRSA from MSSA in ~70 min (Meng et al., 2020). A portable centrifugal 24-chamber LAMP disc, pre-loaded with primers, detected *Mycoplasma pneumoniae, S. aureus*, and MRSA at a LoD of ~10 DNA copies, giving < 1 h results and showing high concordance with PCR.

Emerging CRISPR-based microfluidic assays promise even greater analytical sensitivity. For instance, a PCR-Cas12a fluorescence assay detected *bla*_OXA-1_ gene at ~1.25 copies in < 70 min (Tyumentseva et al., 2025). The bCARMEN system combined droplet microfluidics with Cas13 for multiplexed detection of 27 resistance determinants, including *mec*A/*mec*C, *van* genes, *bla*_KPC_, *bla*_NDM-1_, *bla*_VIM_, *bla*_IMP_, *oxa48*-like, *bla*_CTX-M-15_, and *mcr*1, with 100% accuracy. A simplified CARMEN v2 used pre-loaded, lyophilized microarrays and smartphone-based fluorescence readout in < 3 h, highlighting the potential for near-patient testing (Thakku et al., 2022).

Compared to conventional AST, microfluidic systems consistently shorten turnaround to hours rather than days. The QuickMIC platform reached 95.6% essential and 96.0% categorical agreement with broth microdilution, with only 1.0% very major errors and a mean time of 3 h 13 min (Berinson et al., 2024). Similarly, the QMAC-dRAST platform achieved 96.3% categorical agreement, with very major error rates of only 0.7% for Gram-negatives and 2.2% for Gram-positives, delivering susceptibility results within 6–7 h for most blood cultures (Christensen et al., 2021). Miniaturization of assay volumes reduces sample and reagent requirements while maintaining analytical performance (Wu and Mu, 2024; Nguyen et al., 2023; Azizi et al., 2021, 2018), and the ability to confine single bacterial cells within microchambers or droplets enables the detection of rare resistant subpopulations that might be overlooked by bulk culture methods (Kandavalli et al., 2022). Parallelization and on-chip concentration gradients enable simultaneous multi-drug testing and rapid MIC determination (Wat et al., 2025; Nguyen et al., 2023; Azizi et al., 2021), while compact cartridge-based formats further support point-of-care implementation (Bryant et al., 2020; Meng et al., 2020; Huang et al., 2017).

Despite these advantages, several challenges hinder widespread clinical adoption. Processing of raw clinical samples on-chip is difficult, and incomplete integration of filtration or enrichment steps risks clogging and biofouling (Wu and Mu, 2024). Many systems still depend on external pumps, precision controllers or advanced imaging, which adds operational complexity and cost (Wu and Mu, 2024). Reproducibility and large-scale manufacturing require further optimization, and reliance on primers, probes, or antibodies can restrict pathogen coverage and raise consumable costs. Future designs must focus on robust, multiplexed, and flexible assays to maximize clinical utility (Thakku et al., 2022; Kaprou et al., 2021; Zhang et al., 2018).

### 4.2 Optical and electrochemical biosensing approaches

Biosensors couple a biological recognition element with a physical transducer to produce a measurable signal, enabling rapid and specific detection of bacterial pathogens and their antibiotic-resistance determinants. Among available formats, optical and electrochemical biosensors are the most extensively investigated for clinical and environmental applications, offering miniaturized, low-sample-input assays that can bypass culture and deliver actionable results on short timescales (Laliwala et al., 2024; Magnano San Lio et al., 2023). From a cost standpoint, an m-LAMP-LFB (lateral flow biosensor) test was estimated at $6.5 in total (~$1 for DNA extraction, ~$3.5 for LAMP, and ~$2 for the lateral flow biosensor strip) (Chen et al., 2020). Biosensors themselves act as detection methods by converting biorecognition events into measurable optical or electrochemical signals, yet many recent formats have expanded into hybrid platforms that incorporate molecular amplification or enzymatic assays.

Optical biosensors operate by detecting changes in light (such as absorbance, fluorescence, or refractive index) resulting from the interaction between a target analyte and an immobilized bioreceptor (Laliwala et al., 2024). These devices can be implemented in label-based formats, which employ colorimetric or fluorescent markers, or label-free configurations that exploit intrinsic optical variations (Magnano San Lio et al., 2023). They combine high sensitivity with real-time monitoring and often avoid nucleic-acid amplification or complex preparation. For example, a SERS-based biosensor captured and detected multiple pathogens, including *E. coli, S. aureus*, and MRSA, from complex matrices in ~30 min with ~65% capture efficiency, and correctly identified MRSA in spiked milk and blood (Wang C. et al., 2018). Similarly, a thin-film optical biosensor directly probed *tuf* , *fem*B, and *mec*A genes in positive blood cultures without amplification: hybridization-induced nanometric thickness changes produced a visible color shift readable without specialized instrumentation, achieving 100% sensitivity and specificity for MRSA/MSSA and coagulase-negative staphylococci within ~90 min (Lindsey et al., 2008). In addition, a plasmonic nanosensor using Cu^2+^ and cysteine-modified AuNPs reached 95.8% sensitivity and specificity with ~3-h time to result (Zhang J. et al., 2024). In terms of cost, Zhang et al. (2020) reported that their automated conductometric sensor platform required an instrument investment of approximately $9,000, while the per-sample consumable cost was < $1.

Electrochemical biosensors, in contrast, transduce a biorecognition event into an electrical signal, typically by measuring current (amperometric sensors), voltage or potential (potentiometric sensors), or impedance (impedimetric sensors) changes at an electrode surface (Laliwala et al., 2024). Their inherent sensitivity, rapid response, and ease of miniaturization make them attractive for point-of-care AMR testing (Kao and Alocilja, 2025). Recent examples span phenotypic and genotypic detection: an integrated dual-channel chip simultaneously measured the virulence marker EspB by electrochemical impedance spectroscopy (LoD: 4.3 ng/ml) and β-lactamase activity by differential-pulse voltammetry (LoD: 3.6 ng/ml), distinguishing resistant from susceptible *E. coli* strains with minimal preparation and short assay time (Gunasekaran et al., 2024). For genotypic targets, a portable LAMP-CRISPR/Cas12a biosensor detected the macrolide-resistance gene *erm*B in wastewater after magnetic-bead extraction and LAMP preamplification; Cas12a trans-cleavage of labeled ssDNA (single-stranded DNA) enabled dual readouts (fluorescence and lateral-flow), with an LoD of 2.75 × 10^3^ copies/μl and on-site usability (Mao et al., 2024). Electrochemical immunochromatographic assays deliver very fast phenotypic results: NG-Test Carba 5 reported ~15 min time to result with 98.7%–100% sensitivity and 100% specificity for carbapenemases across multiple evaluations (Yoon et al., 2021; Jenkins et al., 2020); RESIST-4 O.K.N.V. likewise returned ~15-min results with 94.4%–100% sensitivity and 100% specificity for carbapenemase detection (MacDonald and Chibabhai, 2019; Kolenda et al., 2018). For ultra-low-cost settings, Oeschger et al. (2022) reported that the Bacterial Paper Antibiotic Susceptibility Testing Chip (Bac-PAC) paper-based assay could be manufactured at < $2 per chip, with incubation performed in a rechargeable coffee mug instead of a laboratory incubator, thus eliminating major equipment costs.

Additional innovations include the incorporation of nanozyme-based amplification and dual-recognition strategies for improved sensitivity and specificity. Xing et al. (2025) reported an electrochemical biosensor that employed anti-PBP2a antibodies for MRSA-specific capture in combination with vancomycin for *S. aureus* anchoring, thus enabling precise discrimination between resistant and susceptible strains without complex pretreatment. The use of MXene nanozymes with peroxidase-like activity allowed the catalytic conversion of o-phenylenediamine into electroactive 2,2-diaminoazobenzene, generating amplified signals proportional to MRSA concentration and achieving an LoD of 5.0 CFU/ml. The sensor exhibited excellent reproducibility (1.27%), stability (1.62%), and selectivity. In another example, a label-free impedimetric genosensor for *bla*_CTX-M_ gene in *E. coli* and the *bla*_KPC_ gene in *K. pneumoniae* used disposable screen-printed electrodes functionalized with a AuNP/polypyrrole/vanadium-oxide nanocomposite and 4-aminothiophenol-linked ssDNA probes, achieving a linear range of 10^-6^–0.1 ng/μl and LoDs of 0.5 × 10^-7^ ng/μl for *bla*_CTX-M_ and 1 × 10^-7^ ng/μl for *bla*_KPC_. Specificity was high (negligible cross-reactivity), stability persisted for up to three months, and results in clinical isolates showed >95% agreement with PCR (Mahfouz et al., 2025). Other studies confirmed similar high performance, with optical and electrochemical biosensors achieving diagnostic accuracies above 95% and delivering results in as little as 2–5 min for initial readout (Fang et al., 2023; Bianco et al., 2020).

Taken together, optical and electrochemical biosensors routinely deliver clinically relevant results in ~30–90 min (Wang C. et al., 2018; Lindsey et al., 2008), with LoD from a few colony-forming units per milliliter to low-copy-number nucleic acids (Mahfouz et al., 2025; Xing et al., 2025; Gunasekaran et al., 2024; Mao et al., 2024). Optical platforms offer label-free, real-time analysis and strong multiplexing potential, but may be affected by matrix interference and substrate reproducibility (Taha et al., 2024). Electrochemical systems are highly amenable to miniaturization and multiplex integration, with rapid analysis and excellent sensitivity for AMR testing (Kao and Alocilja, 2025; Mahfouz et al., 2025). Looking ahead, priority areas include on-chip sample preparation, seamless coupling to isothermal amplification and CRISPR-based detection, and packaging into compact, user-friendly devices to enable reliable, rapid, and decentralized testing for both clinical diagnostics and environmental surveillance.

### 4.3 Plasmonic nanomaterials

While plasmonic nanomaterials do not constitute a stand-alone method for antibiotic resistance detection, their inclusion in this chapter is justified by their ability to enhance and complement existing approaches. By providing strong optical signal amplification, enabling amplification-free detection, reducing the need for laborious sample preparation, and supporting miniaturization into portable formats, they significantly expand the applicability of conventional assays. Furthermore, their role as versatile transduction elements allows the seamless coupling of molecular recognition with user-friendly readouts, thereby reinforcing both genotypic and phenotypic diagnostic strategies.

Plasmonic nanoparticles, most commonly gold or silver nanostructures, enable label-free optical transduction via localized surface plasmon resonance (LSPR). By coupling sequence- or activity-specific recognition with nanoparticle aggregation or refractive-index changes, these systems can report resistance determinants rapidly and at low cost.

One straightforward implementation is colorimetric DNA testing for resistance genes. Caliskan-Aydogan et al. (2023) developed a gold-nanoparticle (AuNP) biosensor for the *K. pneumoniae* carbapenemase gene *bla*_KPC_ that operates without PCR. AuNPs coated with a complementary probe remain dispersed (red) when the target is present, but aggregate (blue–purple) if it is absent; the visible shift is quantifiable as a LSPR absorbance change. In clinical isolates, the sensor distinguished *bla*_KPC_-positive from negative strains in under 30 min, with a detection limit near ~2.5 ng/μl genomic DNA (~10^3^ CFU/ml) and reported sensitivity/specificity of 79%/97%. In a related gene-targeted format, Saxena et al. (2022) built a dual-mode AuNP aptasensor for *mec*A in *S. aureus*. Thiol-modified DNA aptamers stabilize the AuNPs in the presence of the target. In its absence, salt-induced aggregation produces a red-to-blue shift and an LSPR change. The assay reached a 0.5 ng/μl LoD within ~20 min and was validated against PCR, underscoring utility in low-resource settings.

Plasmonic assays can also report enzyme activity. A culture-independent platform detected carbapenemase activity as pH-induced AuNP aggregation, identifying resistant *K. pneumoniae, Enterobacter cloacae*, and *Citrobacter freundii* at ≥10^5^ cells/ml in < 3 h using simple pre-concentration and smartphone readouts (Santopolo et al., 2021). Functionalized AuNPs similarly differentiated ESBL from carbapenemase producers within ~2 h with >95% sensitivity/specificity (Nag et al., 2020).

Beyond binary color changes, plasmonic spectroscopy can capture richer interaction signatures. Yu et al. (2023) engineered an surface plasmon resonance (SPR) “chemical-nose” array by functionalizing AuNPs with short peptides. Bacterial interactions produced distinct spectral fingerprints that, with ML classification, identified 12 ESKAPE strains and their resistance phenotypes in < 20 min with ~90% accuracy. Another branch of plasmonic sensing involves SERS, in which metallic nanoparticles amplify the Raman signals of biomolecules, as previously described in this work.

Integrated nanoplasmonics combine sensing with active functions. The RAPIDx platform used photothermal plasmonics for lysis, rolling-circle amplification, and multiplexed detection, reporting genotypes and phenotypic markers in ~45 min (Lee et al., 2025). Related nanomaterials also achieve plasmonic-inspired transduction: platinum nanoparticles (PtNPs) on screen-printed electrodes (< $0.1/test) measured catalase activity and produced complete susceptibility profiles in 45–60 min with AUC = 1 (Li et al., 2025).

Plasmonic assays provide rapid results, portable visual readouts, and straightforward chemistry across both gene-targeted and enzyme/interaction-based detection. Some formats avoid nucleic-acid amplification or culture, allowing direct analysis of minimally processed specimens (Caliskan-Aydogan et al., 2023). Per-test material costs can be very low (Li et al., 2025), and smartphone-based readouts support low-resource deployment (Santopolo et al., 2021). Limitations include matrix effects, non-specific aggregation, lack of standardized cut-offs, and dependence on specialized nano-fabrication or optical instrumentation (SPR/Raman), often requiring ML analysis (Yu et al., 2023; Ciloglu et al., 2021). As systems evolve toward multiplexed arrays and integrated devices, plasmonic nanomaterials are positioned to complement molecular and phenotypic diagnostics with fast, scalable resistance readouts near the point of care.

## 5 Artificial intelligence methods

AI has gained significant attention in recent years, particularly in microbiology and genomics, where it has been increasingly applied to AMR, genome analysis, and drug discovery (Shelke et al., 2023; Suster et al., 2024; Branda and Scarpa, 2024). Traditional methods for AMR detection can be very time-consuming, often requiring up to 72 h to assess bacterial growth in the presence of an antibiotic to determine resistance or susceptibility (López-Cortés et al., 2024). AI models can accelerate this process by efficiently analyzing vast datasets, a crucial advantage in genomics, which involves big data. For example, ML analysis of thousands of genomes revealed highly predictable HGT networks, with especially dense transfer routes for ARGs among human-associated microorganisms, highlighting the potential of AI to uncover complex resistance transmission patterns (Zhou et al., 2021). Beyond improving speed, AI has, in some cases, demonstrated greater accuracy than human experts (Branda and Scarpa, 2024).

**Figure 2:**
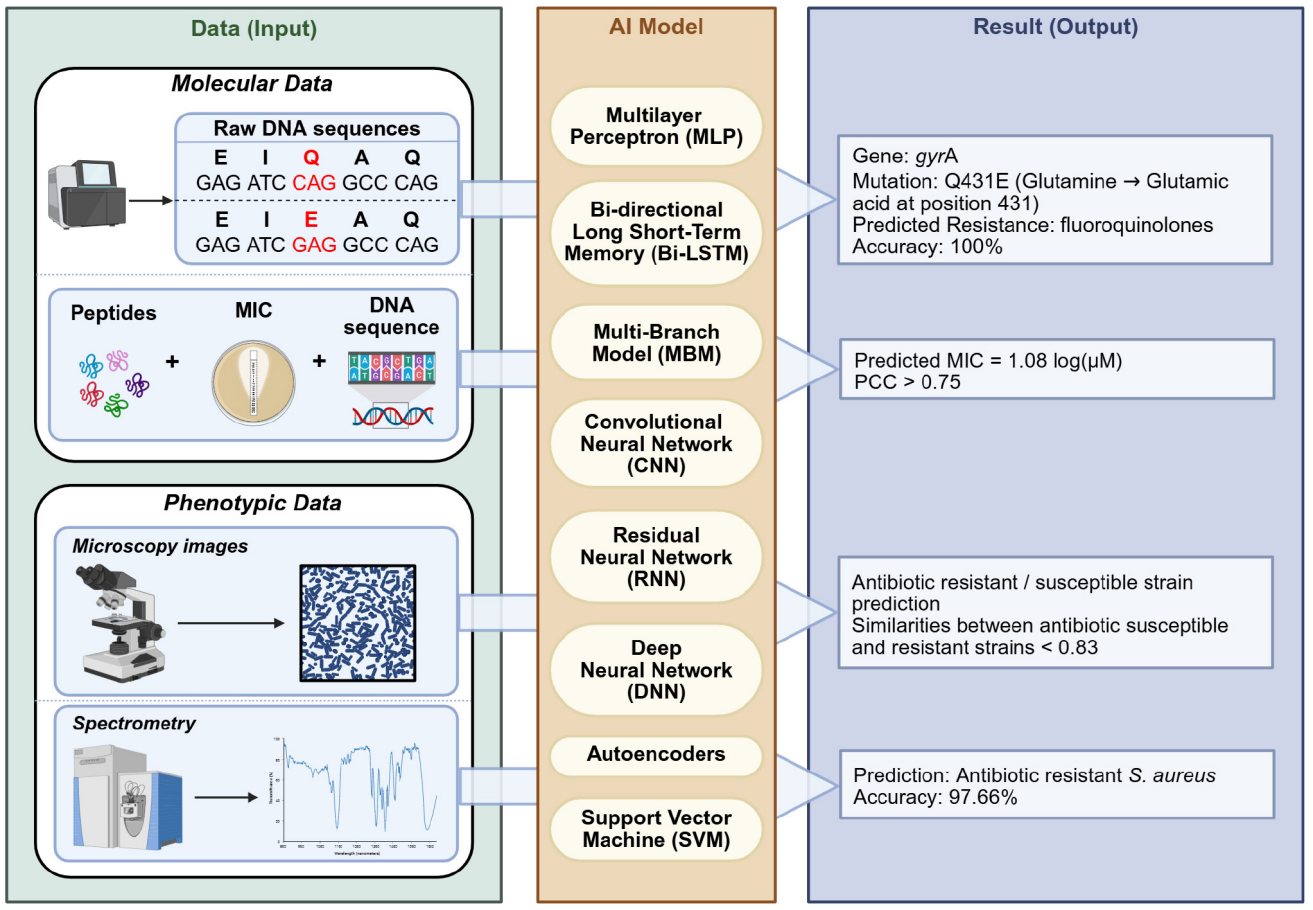
AI-driven analysis of molecular and phenotypic data for AMR prediction. The figure is organized into three panels: **(left)** Data (Input), showing molecular and phenotypic data types used for analysis; **(center)** AI Models, illustrating various machine learning architectures applied to each data type; and **(right)** Result (Output), presenting predicted antimicrobial resistance profiles. The data shown here represent illustrative examples of typical input features and model outputs used in AMR studies. Molecular data include raw DNA sequences, which are preprocessed and analyzed using AI models, including multilayer perceptrons, to identify resistance-associated mutations, such as *gyr*A Q431E conferring fluoroquinolone resistance (Jamal et al., 2020). Separately, antimicrobial peptide sequences, MIC values, and bacterial genomic features can be integrated into deep learning architectures like Bi-LSTM, CNN, and multi-branch models, to predict MIC values against clinically relevant pathogens (Chung et al., 2024). Phenotypic data include microscopy images, processed using CNN or RNN to predict bacterial resistance or susceptibility phenotypes (Ikebe et al., 2024). In addition, spectral data obtained through Raman spectroscopy are analyzed using autoencoders, DNN, or SVM to distinguish resistant from susceptible isolates based on subtle biochemical signatures (Ciloglu et al., 2021). Created in BioRender. Aldea, A. (2025, https://BioRender.com/u5rqf9p).

Given that in AI, data is a critical component, sometimes even more important than the algorithm itself (Bhatt et al., 2024), we categorized AI in AMR applications based on data types: molecular and phenotypic. Figure 2 illustrates this data-driven approach, where raw DNA sequences, antimicrobial peptide (AMP) sequences, MIC values, bacterial genomic features, microscopy images, and spectrometry readings are processed through various AI models to predict resistance or MIC values.

### 5.1 Molecular data

Molecular approaches use DNA sequencing data, such as whole-genome and metagenomic sequences, to predict microbial resistance profiles. AI models can identify complex relationships between genetic features (e.g., mutations, resistance genes, k-mers) and resistance phenotypes, enabling *in silico* susceptibility testing. Numerous studies have investigated the use of AI models for predicting resistance from genomic data (Olatunji et al., 2024). Among these, one of the earliest and most influential studies on AI-driven ARG detection is DeepARG (Arango-Argoty et al., 2018), a deep learning model that significantly improves ARG identification from genomic and metagenomic data, detecting both known and novel ARGs with high accuracy. Another study, HMD-ARG (Li et al., 2021), introduces a multi-task deep learning framework that predicts ARGs, resistance classes, mechanisms, and gene mobility directly from raw protein sequences, unlike DeepARG that depends on reference databases. Building on these, researchers have developed even more advanced architectures. In another study, Pei et al. (2024) introduced ARGNet, which combines an autoencoder with a Convolutional Neural Network (CNN) classifier to identify ARGs without reliance on reference alignment.

Studies have also investigated the prediction of MIC values using ML approaches. Pataki et al. (2020) employed linear regression to predict ciprofloxacin MIC in *E. coli* based on genomic mutations and resistance genes, achieving 65% and 93% accuracy within two- and four-fold dilution ranges, respectively. In related work, Chung et al. (2024) developed an ensemble model incorporating models such as BiLSTM and CNN to predict the MIC of AMPs against *S. aureus, E. coli*, and *P. aeruginosa*, outperforming existing benchmarks. Complementary to these predictive models, Dean et al. (2021) introduced PepVAE (variational autoencoder), a generative approach utilizing a variational autoencoder coupled with antimicrobial activity prediction models to design novel AMPs based on sequence and MIC data, allowing controllable AMP generation with experimental validation.

### 5.2 Phenotypic data

Beyond genomic data, AI is also transforming phenotypic detection of resistance, analyzing how bacteria look, grow, or behave in the presence and absence of drugs. Here, DNNs can process complex data like microscopy images, spectroscopy readouts, or time-series signals that indicate a bacterium's drug response.

Computer vision algorithms can identify antibiotic-resistant bacteria from microscopic images by detecting subtle morphological changes. Hayashi-Nishino et al. (2022) trained a CNN on transmission electron microscope (TEM) images to classify *E. coli* resistance, achieving 94% accuracy. The model detected structural changes like altered cell envelopes and sphericity, linked to genetic resistance. Similarly, Zagajewski et al. (2023) developed a deep learning framework leveraging fluorescence microscopy to segment and classify single-cell phenotypes, predicting antimicrobial susceptibility with 80% accuracy and estimating MIC values within just 30 min. In a different approach, Brown et al. (2020) created an automated optical system integrated with deep learning to accelerate AST, achieving over 90% accuracy in detecting bacterial growth within 7 h.

AI is also being applied to laboratory spectroscopy data to infer resistance (Zhang et al., 2022; Feucherolles et al., 2021). In a study, Lu et al. (2022) combined confocal Raman microspectroscopy with a deep residual neural network (ResNet) to classify *K. pneumoniae* isolates, identifying whether they carried certain resistance genes or had resistant phenotypes. Similarly, mass spectrometry, a widely used tool in clinical microbiology labs, is now being used for resistance prediction. A recent study introduced MSDeepAMR (López-Cortés et al., 2024), which enhances MALDI-TOF mass spectrometry by using deep learning to predict antibiotic resistance from raw spectra, achieving high accuracy (over 0.83 AUC) and up to 20% performance gains with transfer learning. This approach enables real-time AMR detection in clinical settings.

### 5.3 Data representation and challenges in AI for AMR prediction

AI models for AMR prediction rely on how genomic sequences are numerically represented before being processed in the learning pipeline. Different strategies have been implemented, such as constructing dissimilarity or bit-score matrices relative to known resistance genes, encoding raw protein or nucleotide sequences with one-hot representations, or generating latent embeddings through autoencoders. These approaches enable the identification of antibiotic resistance genes and support the detection of both known and previously uncharacterized determinants (Pei et al., 2024; Li et al., 2021; Arango-Argoty et al., 2018). Spectral data such as Raman and MALDI-TOF profiles are commonly preprocessed through baseline correction, smoothing, binning, and peak extraction, with some studies also applying wavelet-based feature detection. These reduced representations are then used as input for ML models, where CNNs or ensemble classifiers can learn discriminative patterns for antimicrobial resistance prediction (López-Cortés et al., 2024; Feucherolles et al., 2021; Zhang et al., 2022; Lu et al., 2022). Image-based datasets, such as transmission electron microscopy images of bacterial cells, require preprocessing steps including normalization, segmentation, and data augmentation before being analyzed with convolutional neural networks. These models can then capture subtle morphological signatures associated with resistance phenotypes (Hayashi-Nishino et al., 2022). Collectively, these preprocessing strategies ensure that heterogeneous data sources (genomes, spectra, and images) can be systematically featurized and exploited by ML algorithms.

While AI has significant potential to advance AMR research, several limitations must be addressed. First, there is a need for large, high-quality, and diverse datasets, as models developed from insufficient or biased data are prone to inaccuracies and limited generalizability (Mohammed et al., 2025). The growing emphasis on data-centric AI highlights that model performance is often constrained more by data quality than by algorithmic advances. Second, the issue of interpretability remains a significant concern, with many AI systems functioning as “black boxes” that limit transparency and reduce confidence in their clinical applicability Li X. et al. (2022). Emerging approaches such as attention mechanisms, feature attribution methods, and self-interpretable models attempt to mitigate this challenge, but widespread clinical trust is still lacking. Finally, the ability of AI models to generalize across diverse bacterial populations and sequencing or spectroscopic platforms remains a pressing concern, since variations in genomic backgrounds and technological methods can undermine reproducibility and translational relevance (López-Cortés et al., 2024; Olatunji et al., 2024; Lu et al., 2022). Transfer learning strategies, where models trained on one dataset are adapted to external cohorts, have shown promise in partially addressing this limitation (López-Cortés et al., 2024).

Despite these challenges, continued progress in data preprocessing, interpretability research, and standardized benchmarking is gradually strengthening the role of AI in AMR detection.

## 6 Comparative overview of antibiotic resistance detection methods and their applicability in low-resource settings

**Figure 3:**
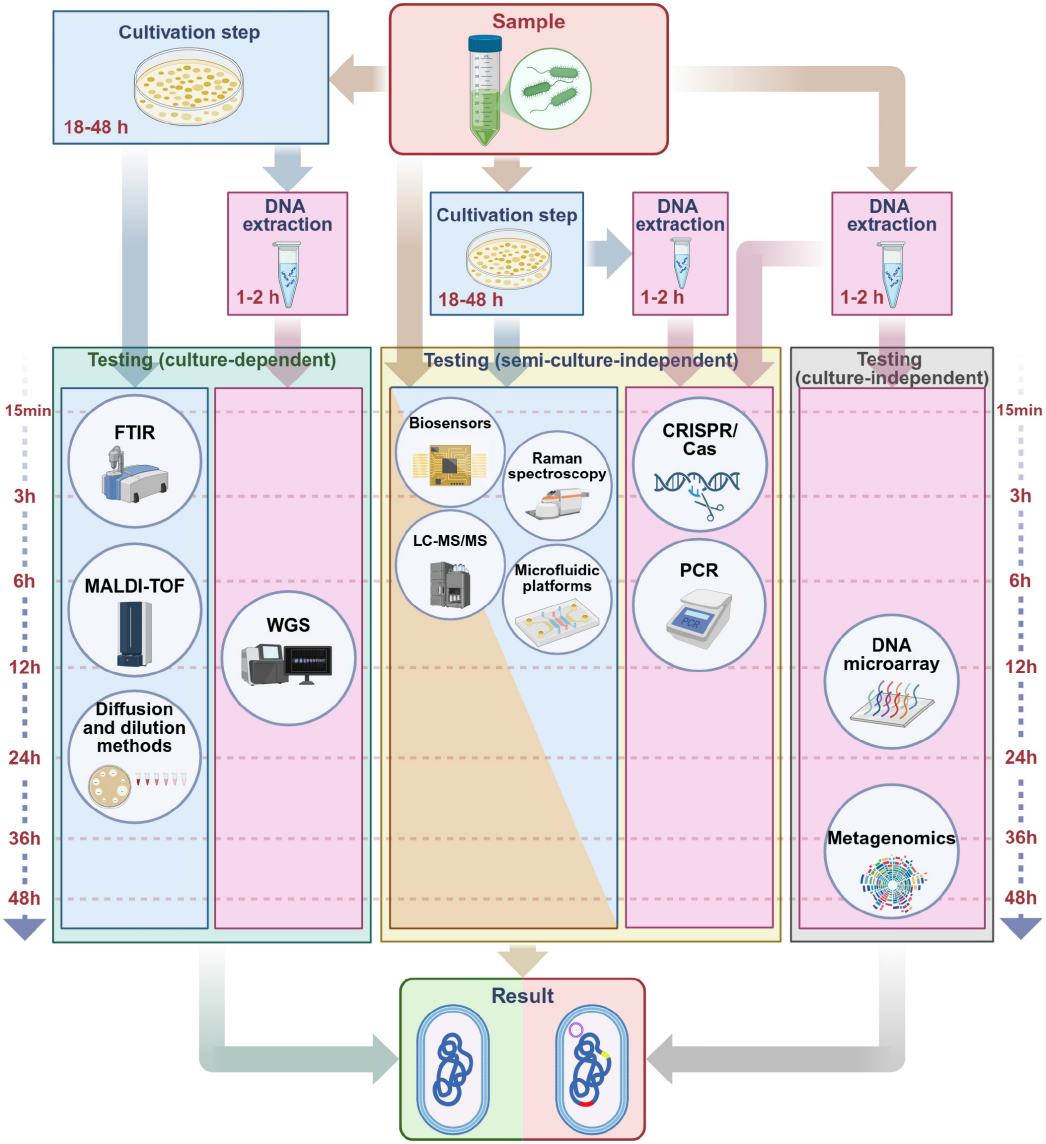
Comparative overview of antibiotic resistance detection methods by culture dependency and time to result. This figure provides a schematic comparison of major categories of antibiotic resistance detection methods and their indicative timeframes. Where applicable, an upstream DNA extraction module (1–2 h) is shown, preceding nucleic-acid-based workflows (PCR, DNA microarray, WGS, metagenomics, and CRISPR/Cas). **(Left)** Culture-dependent methods rely on an obligatory cultivation step of 18–48 h prior to testing. These include traditional phenotypic assays, FTIR spectroscopy, MALDI-TOF MS, and whole genome sequencing, which subsequently yield results within 1–24 h. **(Center)** Semi-culture-independent methods, exemplified by biosensors, CRISPR/Cas, Raman spectroscopy, LC-MS/MS, PCR, and microfluidic platforms, can be implemented with or without prior enrichment depending on the protocol, providing results within 15 min–7 h. **(Right)** Culture-independent methods bypass growth entirely, directly analyzing molecular signatures from the sample. These include DNA microarrays and metagenomics, with typical times to result ranging from 7 to 48 h. Arrows indicate the general workflow, highlighting how diagnostic speed improves as culture requirements are reduced, from multi-day phenotypic assays to near real-time molecular or biosensor-based approaches. Created in BioRender. Aldea, A. (2025, https://BioRender.com/z40p4eu).

To provide a consolidated view of the key characteristics of antibiotic resistance detection methods, Table 2 presents a comparative synthesis of culture requirements, turnaround time, sensitivity, specificity, costs, and main limitations. All values were compiled from peer-reviewed studies cited in the corresponding method sections of this review, and represent ranges reported across different pathogens, sample types, and evaluation protocols. In addition, Figure 3 offers a complementary perspective by mapping these approaches according to their reliance on bacterial culture and their indicative turnaround times. This schematic emphasizes the major conceptual divide between culture-based assays, which inherently delay diagnosis, and culture-independent technologies, which can provide actionable information within hours. Together, the Table 2 and Figure 3 underscore the trade-offs between speed, accuracy, infrastructure needs, and cost-efficiency across detection strategies.

**Table 2:** Comparative summary of antibiotic resistance detection methods.

**Method**	**Culture requirement**	**End-to-end time to result^a, b^**	**Sensitivity^c^**	**Specificity^c^**	**LoD**	**Cost for equipment**	**Cost per test^d^**	**Main limitations**
Diffusion and dilution-based methods	Required	Up to 48 h	~95%–100%	~95%–100%	N/A	~$12,000–$42,500	~$1.50–$50	Require culture facilities and incubators; need trained personnel for standardized interpretation; incubation time slows clinical decision-making; require multiple plates/microtiter systems and biosafety infrastructure.
Raman spectroscopy	Optional	~1.5–3 h/ ~24 h	~96%–100%	~85%–100%	~10^3^–10 CFU/ml	~$5,000–$400,000	~$0–$25	High upfront equipment cost; SERS substrates costly and variable; analysis requires chemometrics/machine learning expertise; reproducibility challenges hinder standardization; reagent supply and infrastructure restrict Low- and Middle-Income Countries (LMICs) adoption.
MALDI-TOF mass spectroscopy	Required	Up to 24 h	~99%–100%	~99%–100%	~10^5^–10^3^ CFU/ml	~$200,000–$500,000	~$0.20–$7	High capital cost and annual maintenance; requires pure cultures and biosafety infrastructure; skilled operators needed; limited availability in LMICs despite low per-sample consumable cost
LC-MS/MS	Optional	~ 3 h/~ 24 h	~96%–100%	~100%	~10^7^–10^3^ CFU/ml	~$75,000–$500,000	–	Very high equipment cost; complex sample preparation requiring trained proteomics staff; reagents and columns expensive; low feasibility for LMICs.
FTIR	Required	Up to 24 h	~74%–99%	~66%–94%	~10^5^–10^3^ CFU/ml	~$15,000–$150,000	~$0–$4	Requires culture before testing; spectra interpretation depends on machine learning/databases; access to instruments and expertise limited in LMICs; substrates add recurring costs.
PCR-based assays	Optional	5–7 h/ ~24 h	~46%–100%	~95%–100%	~10^4^–10^2^ DNA copies for PCR, ~100–10 DNA copies for qPCR, ~2–1 DNA copies for dPCR	~$750–$200,000	~$0.22–$10	Require nucleic acid extraction kits; qPCR/dPCR instruments costly and not always accessible; demand molecular expertise for optimization; reagents can be expensive and supply-chain dependent.
DNA microarray	Not required	7–8 h	~93%–100%	~83%–100%	From 10^2^–10^1^ to ~30 DNA copies/μl	~$10,000–$150,000	~$40–$400	Require specialized scanners, fluorescent labeling kits, and bioinformatics pipelines; constrained to predefined gene panels; high per-sample consumable costs; limited adoption in LMICs.
Metagenomics	Not required	Up to 48 h	~61%–100%	~64%–100%	N/A	~$3,000–$1,000,000 only for the sequencer	~$130–$685	Infrastructure-intensive; high per-sample cost; requires advanced bioinformatics; complex workflows limit feasibility in LMICs; consumables often unaffordable.
WGS	Required	From 7–9 h to 2–5 days	~87%–99.6%	~97%–98.4%	N/A	~$3,000–$1,000,000 only for the sequencer	~$50–$238	Require expensive sequencers, high-throughput computing, and specialized bioinformatics expertise; library prep kits and sequencing reagents costly and often unavailable in LMICs.
CRISPR/Cas-based detection	Optional	2–4 h/~24 h	~96.5%–100%	~100%	~10^3^–10 DNA copies	~$1,900–$60,000	~$12	Depend on nucleic acid extraction and pre-amplification; CRISPR enzymes and gRNAs remain costly and less available in LMICs; assays require molecular handling skills; limited standardization across platforms.
Microfluidic platforms	Optional	~30 min–7 h	~97.7–100%	~78.8–93.2%	~10^5^ CFU/ml; ~10–1 DNA copies	Low (application-dependent)	< $0.3	Many systems require pumps/controllers or custom fabrication; reproducibility and scaling remain barriers; risk of channel clogging and biofouling when processing complex samples.
Biosensors	Optional	~15 min–3 h	~94.4%–100%	~95.2%–100%	~5 CFU/ml; ~10^3^ DNA copies/μl; ~4 ng/ml (protein/enzyme targets)	Low (application-dependent)	~$1–$6.5	Substrate/electrode reproducibility issues; need for specific antibodies/probes increases cost and dependency on supply chains; many platforms are prototypes with limited standardization; specialized readers may be costly for LMICs.

^a^For culture-dependent methods, the reported time to result includes both the incubation period recommended by European Committee on Antimicrobial Susceptibility Testing (EUCAST) (2025) (18 h for most bacterial species). For molecular methods, the DNA extraction step (1–2 h) is also included.

^b^For semi-culture-independent methods, results are shown as dual values (with/without culture).

^c^Values are reported as ranges summarizing several independent studies, since sensitivity and specificity varied depending on pathogen and experimental setup.

^d^Estimated prices refer to general analyses and are not specific to assays dedicated to antibiotic resistance detection.

Traditional phenotypic approaches, such as diffusion and dilution-based assays, continue to serve as standard tools in clinical microbiology owing to their operational simplicity and low cost. They generally achieve near-perfect sensitivity and specificity, require only basic incubators and consumables, and remain among the most affordable methods, making them particularly suitable for implementation in low-resource settings. Practical examples include disk diffusion, E-test strips, and broth microdilution on reusable plates, together with low-cost adaptations such as portable mini-incubators for field or decentralized laboratories (Klyusko et al., 2025; Talebipour et al., 2024). However, their relatively long turnaround times limit their clinical utility, especially in time-sensitive or complex infections (Gajic et al., 2022). Although phenotypic assays can reveal certain resistance mechanisms [e.g., ESBL (Hombach et al., 2017) or carbapenemase production (Cimen et al., 2025)], they cannot provide a complete picture of the underlying genetic basis.

In contrast, molecular methods, including PCR, offer rapid and accurate detection with high sensitivity and specificity, though their reliance on prior knowledge of target genes restricts their capacity to detect novel or unexpected resistance variants (Vogelstein and Kinzler, 1999; Heid et al., 1996). PCR-based assays typically deliver results in under a day and remain relatively inexpensive per test, although equipment and reagent costs vary widely. From a technical standpoint, modern PCR platforms are characterized by very low LoD values, with digital PCR capable of reliably identifying down to a single DNA copy. To address constraints in low-resource environments, isothermal amplification strategies have been integrated into portable or paper-based diagnostic platforms, often operating with lyophilized reagents (eliminating cold-chain requirements) and producing colorimetric or fluorescence signals interpretable by eye or via smartphone imaging (Mao et al., 2024; Thakku et al., 2022; Santopolo et al., 2021; Meng et al., 2020; Huang et al., 2017). Building on this principle, CRISPR/Cas-based systems coupled with isothermal amplification have also been developed into low-cost, field-deployable formats, enabling multiplexed resistance gene detection with readouts accessible through lateral-flow strips or smartphones (Mao et al., 2024; Thakku et al., 2022; Santopolo et al., 2021). Coupled with pre-amplification, CRISPR/Cas diagnostics can achieve limits of detection of only a few DNA copies, supporting their use in early detection under low bacterial load conditions.

DNA microarrays enable the parallel screening of multiple genes, yet they are similarly constrained by this dependence on predefined sequences (Brazelton de Cardenas et al., 2021; Lu et al., 2014). Although they report high sensitivities and specificities, their cost per assay and need for specialized scanners limit widespread adoption. Their reported LoDs vary from a few dozen down to as few as 10 DNA copies per microliter, which illustrates their analytical power but also emphasizes dependence on sample purity and hybridization efficiency. Genomic tools such as WGS and metagenomics offer comprehensive insights into the resistome. These techniques enable the identification of both known and emerging resistance determinants, but are currently limited in clinical practice by high costs, longer processing times, and the need for bioinformatic infrastructure (Le et al., 2024; Greninger, 2018; Wyres et al., 2014). Unlike other methods, WGS and metagenomics do not define LoD in absolute terms, as their detection capacity depends primarily on sequencing depth, read coverage, and data analysis pipelines rather than on minimal input concentrations. To overcome these barriers, collaborative regional sequencing hubs based on a hub-and-spoke model have been developed. In this approach, centralized facilities with high-throughput sequencers, bioinformatic resources, and trained personnel support multiple peripheral hospitals and clinics serving as sample collection and referral centers. This model distributes costs, harmonizes protocols, and expands access to genomic surveillance. Examples already exist: the Africa CDC's Africa Pathogen Genomics Initiative (Africa PGI), launched in 2020, has already established 13 regional Centers of Excellence across the continent (Africa Centres for Disease Control and Prevention, 2020b). Moreover, under Africa PGI 2.0, additional regional coordination centers are being implemented (Africa Centres for Disease Control and Prevention, 2020a), alongside collaborations such as the Africa CDC-Illumina partnership, which aims to equip laboratories in up to 25 countries with operational NGS capacity by the end of 2025 (Illumina and Africa Centres for Disease Control and Prevention, 2025).

Although WGS is used in some reference laboratories, especially for pathogens like *Mycobacterium tuberculosis* (Meehan et al., 2019), its broader implementation remains restricted. Spectroscopy-based methods such as Raman spectroscopy, MALDI-TOF MS, LC-MS/MS, and FTIR provide rapid, culture-based or culture-independent analysis. Raman and FTIR are faster but face reproducibility and specificity challenges, MALDI-TOF offers high accuracy at low per-test costs but requires costly equipment and pure cultures, while LC-MS/MS delivers excellent accuracy but involves complex workflows and expensive reagents. Their clinical use remains limited by requirements such as pure cultures (MALDI-TOF) or signal reproducibility (Raman, FTIR), highlighting the need for further validation before routine implementation (Suleiman et al., 2022; Ciloglu et al., 2021; Idelevich et al., 2018; De Bruyne et al., 2011).

Newer technologies, including CRISPR/Cas-based detection systems and AI-assisted diagnostic platforms, represent emerging tools in the field. CRISPR-based diagnostics allow rapid and specific identification of resistance genes (Agha et al., 2025; Lai et al., 2025), but are still in early development and not yet standardized for clinical use. They demonstrate excellent reported sensitivity and specificity, but remain hindered by reagent costs and the need for pre-amplification steps. Moreover, portable microfluidic chips with lateral-flow or smartphone-based readouts illustrate their potential for resource-limited deployment, with reported LoDs as low as a few CFU/ml or nanogram protein levels. Despite this ultra-sensitivity, reproducibility and standardization remain critical challenges before clinical translation (Mao et al., 2024; Thakku et al., 2022; Meng et al., 2020; Huang et al., 2017).

AI-based approaches can support data interpretation and resistance prediction, particularly when applied to large-scale genomic or spectroscopic data (Chung et al., 2024; Pei et al., 2024; Arango-Argoty et al., 2018). Nevertheless, their performance depends heavily on the availability of high-quality training datasets (Lewin-Epstein et al., 2021), and most applications are currently limited to research settings.

In addition, the diagnostic performance of each method depends not only on the technology itself but also on the type of sample analyzed, which can substantially influence turnaround time, sensitivity, and specificity. Reported LoD values should also be considered with caution, as they are strongly influenced by sample pretreatment steps such as nucleic acid extraction, removal of inhibitors, or prior culture enrichment.

Taken together, the evidence summarized in Table 2 and Figure 3 highlight the central trade-offs across methods. Classical phenotypic methods remain practical and inexpensive but are constrained by slow turnaround times. Targeted molecular approaches, such as PCR, deliver rapid and accurate results yet cannot capture unknown resistance determinants. Untargeted genomic tools (WGS, metagenomics) provide the most comprehensive resistome profiles and are valuable for surveillance and discovery, though their broader use is still limited by costs and infrastructure. Spectroscopy-based methods offer rapid analysis but require further standardization to ensure reproducibility. AI-assisted platforms add predictive power and enable integration of heterogeneous datasets, but depend on large, high-quality training data. These contrasts determine context-specific suitability: phenotypic and PCR-based assays remain central to routine diagnostics, WGS and metagenomics are best suited for surveillance and research, spectroscopy and MALDI-TOF fit centralized laboratories, while CRISPR, microfluidics, and biosensors hold particular promise for decentralized and low-resource settings.

## 7 Outlook

The growing diversity of methods available for detecting antibiotic resistance offers opportunities to improve diagnostic speed, accuracy, and accessibility across diverse settings. Each category of methods: phenotypic assays, molecular tools, spectrometry-based platforms, biosensors, microfluidic systems, and AI approaches brings unique strengths, but no single approach is sufficient on its own. The most promising directions point toward integrated diagnostic platforms that combine complementary methods into accessible, scalable, and context-specific devices. Synergistic combinations include spectroscopy with AI for rapid pathogen identification and resistance prediction, microfluidics coupled with isothermal amplification and CRISPR-based detection for portable high-sensitivity assays, biosensors merged with plasmonic or electrochemical transduction elements, and metagenomic sequencing complemented by targeted PCR for comprehensive surveillance with rapid confirmation. These integrations not only enhance analytical performance but also align with diverse needs: fast turnaround in hospitals, broad surveillance during outbreaks, and affordability in low-resource environments. Several proof-of-concept platforms already demonstrate this potential, including microfluidic chips with smartphone readouts (Thakku et al., 2022; Wu et al., 2018), paper-based assays that integrate phenotypic growth monitoring with colorimetric biosensors (Oeschger et al., 2022), or plasmonic nanomaterials for amplification-free gene detection (Caliskan-Aydogan et al., 2023). Such convergence signals the trajectory toward the next generation of implementable diagnostic platforms.

Despite substantial progress, methodological inconsistencies, particularly in primer selection and PCR protocols, continue to complicate data comparability and hinder broader ecological interpretations. As a step toward harmonization, we provide a curated list of primer pairs, validated against gene sequences in the CARD database (Alcock et al., 2023), which may serve as a resource for future assay design and benchmarking.

AI applications, though still nascent, have the potential to complement both phenotypic and molecular frameworks by accelerating data analysis and enhancing detection accuracy. Their integration will depend not only on computational performance but also on the interpretability, generalizability and clinical validation of resulting models.

Looking ahead, the most impactful strategies will be those tailored not only to specific scientific or clinical questions, but also to resource constraints, enabling resistance monitoring in remote ecosystems, decentralized hospitals, and field laboratories. Only through such integration and standardization can fragmented detection efforts evolve into a comprehensive, One Health-oriented surveillance of antibiotic resistance.
